# The autism-linked gut microbial metabolite *p*-cresol inhibits host catecholamine biosynthesizing enzymes to elicit social deficits

**DOI:** 10.1038/s42003-025-09207-0

**Published:** 2025-11-22

**Authors:** Geoffroy Mallaret, Juliette Canaguier, Jacques Callebert, Nicolas Caramello, David Fabregat-Safont, Nicolas Glaichenhaus, Oscar J. Pozo, Jean-Marie Launay, Laetitia Davidovic

**Affiliations:** 1 Institut de Pharmacologie Moléculaire et Cellulaire, Université Côte d’Azur, CNRS, INSERM, Valbonne, France; 2 Service de Biochimie et Biologie Moléculaire, INSERM U942, Hôpital Lariboisière, AP-HP, Paris, France; 3 Université Grenoble Alpes, CNRS, CEA, Institut de Biologie Structurale (IBS), Grenoble, France; 4 Applied Metabolomics Research Group, Hospital del Mar Research Institute, Barcelona, Spain; 5 Environmental and Public Health Analytical Chemistry, Research Institute for Pesticides and Water (IUPA), University Jaume I, Castelló, Spain; 6 Alliance FondaMental, Créteil, France

**Keywords:** Social behaviour, Enzyme mechanisms

## Abstract

Autism spectrum disorder (ASD) is associated with altered gut microbiota and elevated levels of the microbial metabolite *p*-cresol. We previously demonstrated that -cresol induces social deficits in male mice, alongside reduced excitability of dopamine neurons in the ventral tegmental area, a key catecholamine region in the reward circuit known to control social behavior. Here, we explore the molecular mechanisms underlying these effects. We investigated *p*-cresol and its host conjugate, *p*-cresol sulfate, biodistribution in peripheral and central matrices. We show that both metabolites accumulate in the brainstem and impair catecholamine biosynthesis by inhibiting tyrosine hydroxylase (TH) and dopamine-β-hydroxylase (DBH). In silico docking predicts competitive binding of both metabolites to the catalytic pockets of TH and DBH. DBH inhibition alone was sufficient to recapitulate *p*-cresol-induced social deficits. These findings identify inhibition of host enzymes as a mechanism by which microbial metabolites alter brain function and behavior, linking gut microbiota to ASD-relevant social impairments.

## Abstract (web summary)

The microbial metabolite *p*-cresol and p-cresol sulfate accumulate in brainstem and inhibit TH and DBH, key enzymes in catecholamine synthesis. This disruption alters brain function and behavior, with DBH inhibition reproducing p-cresol-induced social deficits linked to ASD.

## Introduction

Autism spectrum disorders (ASD) are a group of common neurodevelopmental disorders that arise from complex interactions between individual genetic susceptibility and exposure to adverse environmental factors^1^. ASD is characterized by a heterogeneous clinical presentation, with core symptoms including deficits in social interaction and communication, repetitive or stereotyped behaviors, restricted interests, and atypical sensory processing^2^. Common comorbid conditions include attention deficits and hyperactivity disorder (ADHD) as well as anxiety disorder^2^. The global median prevalence of ASD approaches 1%, with an increasing incidence reported in recent decades^3^. While ~10–25% of ASD cases can be attributed to mutations in specific genetic *loci*, evidence from twin studies underscores an equal contribution of genetic and environmental factors to ASD risk, suggesting that non-genetic influences may play a significant role in disease onset and progression^4^.

Among these environmental factors, growing evidence supports the involvement of the gut microbiota and its metabolic byproducts in ASD pathophysiology^1,5^. Clinical studies have consistently reported a higher incidence of gastrointestinal (GI) symptoms in ASD patients compared to neurotypical individuals, as well as significant alterations in gut microbiota composition^6–10^. These microbial imbalances are often accompanied by dysregulated microbiota-derived aromatic metabolites, including tryptophane-derived indoles and phenylalanine/tyrosine-derived compounds^5,11–13^. Notably, similar patterns of gut microbiota dysbiosis and metabolite alterations have been observed across multiple ASD mouse models, reinforcing the idea of a microbiota-brain connection in ASD^14–19^.

Among the microbiota-derived metabolites implicated in ASD, *p-*cresol (*para*-cresol, 4-cresol, 4-methylphenol) has emerged as a particularly relevant candidate to study due to its neuroactive properties and consistent elevation in ASD patients^11–13^. Using targeted and untargeted metabolomic approaches, increased levels of *p*-cresol and/or of its abundant host conjugated form, *p-*cresol sulfate (4-cresol sulfate, *para*-cresol sulfate) and minor conjugate *p-*cresol glucuronide, have been consistently detected in the urine and feces of individuals with ASD, suggesting altered microbial metabolism in these individuals^20–30^. *p*-Cresol is produced by bacterial fermentation of dietary tyrosine, primarily occurring in the colon in humans and in the cecum/colon in mice, where its local concentrations can reach the high micromolar range^31,32^. However, its systemic and central bioavailability remains unclear, as *p*-cresol undergoes rapid intestinal and hepatic conjugation by aryl sulfotransferases, forming *p*-cresol sulfate, which is subsequently excreted in urine^32^. *p-*Cresol sulfate appeared undetectable in the serum of germ-free mice, further ascertaining the microbial origin of its precursor *p*-cresol^33^. Until recently^34^, the low abundance of *p*-cresol in blood or tissues has made it difficult to determine to which extent free *p*-cresol enters systemic circulation or crosses the blood-brain barrier to exert direct effects on the central nervous system.

In a previous study, we showed that chronic exposure to *p*-cresol from mid-gestation to weaning in the offspring of pregnant dams chronically treated with *p*-cresol in drinking water had minimal effects on early development in mice, but led to persistent impairments in adult social behavior as well as stereotypies^35^. In a separate study, we reported similar social behavior deficits and stereotyped behavior in mice exposed chronically to *p*-cresol in drinking water starting at 4-weeks of age, corresponding to the juvenile-adolescent stage-continuing to adulthood^36^. Notably, neither exposure paradigm affected locomotor or exploratory activity or induced anxiety-like behaviors, suggesting that *p*-cresol selectively induced ASD-like behaviors, but not behaviors linked to common ASD comorbid conditions (i.e., ADHD or anxiety). In the post-weaning paradigm, which models sustained GI exposure to *p*-cresol, we also observed gut microbiota dysbiosis and elevated fecal *p*-cresol levels^36^. We also linked *p*-cresol-induced social impairments to a decrease in the excitability of ventral tegmental area (VTA) dopamine (DA) neurons, a key component of the social reward circuitry, highlighting potential catecholamine dysfunction^36^. However, the precise mechanisms by which *p*-cresol contributes to neurobehavioral deficits remain unknown. In particular, it is unclear whether these effects result from systemic exposure to *p*-cresol and its conjugates or whether *p*-cresol exerts a direct action on the brain by disrupting catecholamines or their signaling.

In this study, we sought to address these questions by systematically assessing in *p*-cresol-treated mice the biodistribution of *p*-cresol and its main conjugate *p*-cresol sulfate across various biological matrices. Additionally, we investigated the impact of *p*-cresol on DA and noradrenaline (NA) as well as their biosynthesis enzymes, in relation with social behavior.

## Results

### *p-*Cresol exposure increased levels of *p-*cresol and its main host conjugate *p-*cresol sulfate in feces, urine, plasma, and brainstem

**Fig. 1:**
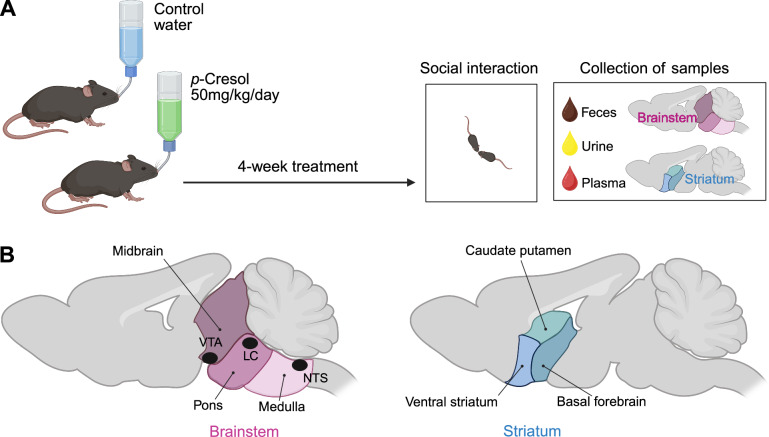
Experimental design. **A** Timeline **B** Brain areas used in this study. VTA Ventral Tegmental Area, LC locus coeruleus, NTS Nucleus Tractus Solitarius. Created in BioRender. Davidovic, L. (2025) https://BioRender.com/htkrhhk.

**Fig. 2:**
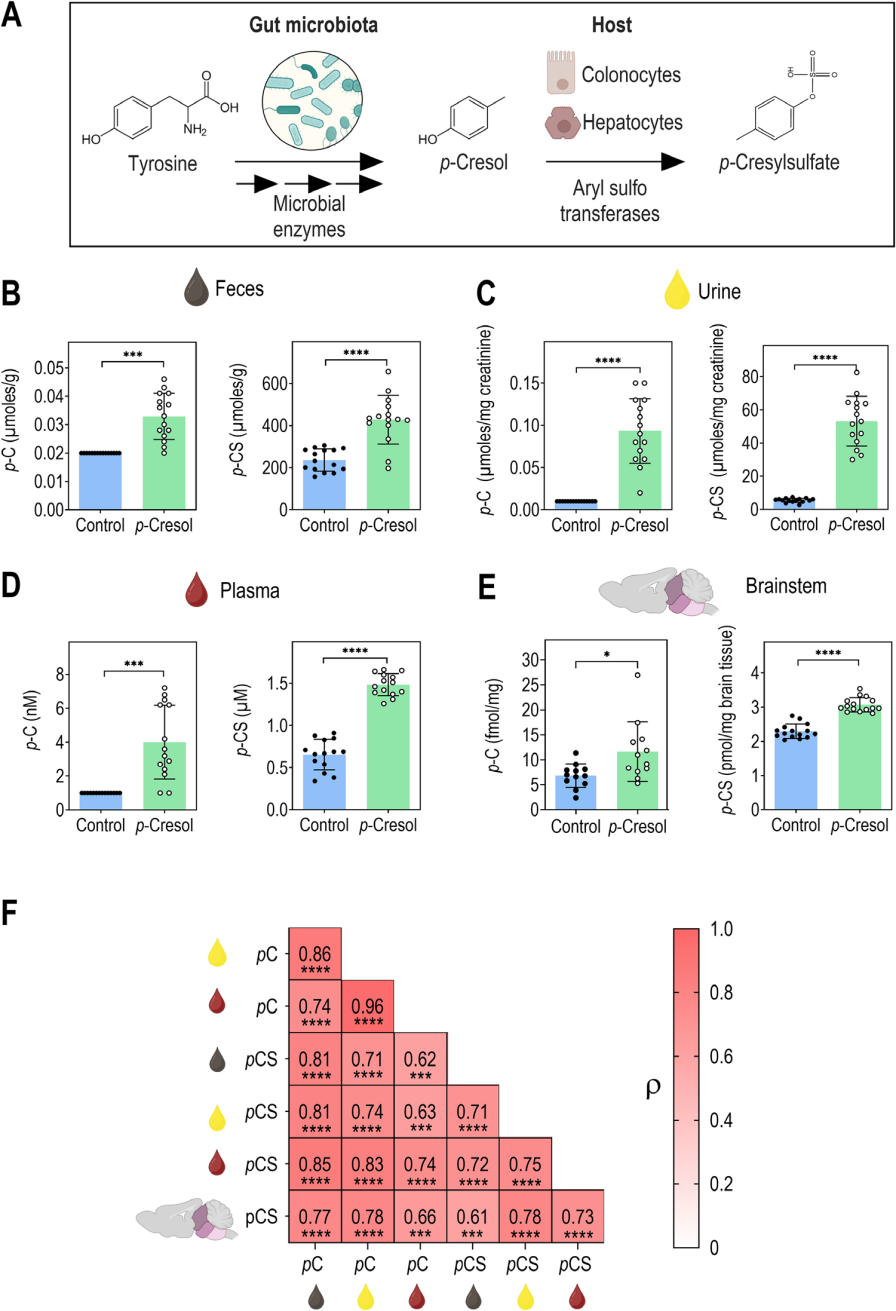
Mice exposed to *p*-cresol show increased levels of *p*-cresol and *p*-cresol sulfate in feces, urine, plasma, and brainstem. **A** *p*-Cresol and *p*-cresol sulfate biosynthesis pathway. **B** Fecal levels of *p*-cresol and *p*-cresol sulfate. One sample *t* test vs. Limit of Detection (LOD) = 0.02: *t* = 6.128, df = 14, ****p < 0.0001; Unpaired *t* test: *t* = 5.838, df = 28, ****p < 0.0001; respectively. **C** Urine levels of *p*-cresol and *p*-cresol sulfate. One sample *t* test vs. LOD = 0.01: *t* = 8.427, df = 14, ****p < 0.0001; Unpaired *t* test: *t* = 12.32, df = 28, ****p < 0.0001; respectively. **D** Plasma levels of *p*-cresol and *p*-cresol sulfate. One sample *t* test vs. LOD = 1.00: *t* = 5.143, df = 13, ***p = 0.0002; Unpaired *t* test: *t* = 13.82, df = 26, ****p < 0.0001; respectively. **E** Brainstem levels of *p*-cresol and *p*-cresol sulfate. Unpaired *t* tests: *t* = 2.607, df = 22, *p = 0.0161; *t* = 10.13, df = 28, ****p < 0.0001; respectively. **F** Correlations between *p*-cresol and *p*-cresol sulfate levels in feces (brown drop), urine (yellow drop), plasma (red drop), and brainstem (*n* = 30 (*n* = 15 Control, *n* = 15 *p*-Cresol) except for *p*-cresol levels in plasma, *n* = 28 (*n* = 14 Control, *n* = 14 *p*-Cresol)). Spearman’s rank ρ correlation coefficients test: ***p < 0.001, ****p < 0.0001. **B**–**E** *n* = 15 Control, *n* = 15 *p*-Cresol, except: plasma *p*-cresol and *p*-cresol sulfate: *n* = 14 Control, *n* = 14 *p*-Cresol, brainstem *p*-cresol: *n* = 12 Control, *n* = 12 *p*-Cresol. Data are presented as dot plots showing means ± standard deviation. **F** Heatmap of pairwise Spearman’s rank ρ correlation coefficients between variable pairs.

*p-*Cresol being derived from the bacterial degradation of tyrosine by the gut microbiota, natural exposure occurs mainly via the GI tract. To mimic GI exposure, C57BL/6J male mice were exposed to *p-*cresol dispensed in drinking water (Fig. 1A), as previously described^36^. Luminal microbial *p*-cresol is then mainly conjugated by the host into *p*-cresol sulfate by aryl sulfotransferases present in colonocytes and in hepatocytes (Fig. 2A). However, the relationships between *p-*cresol and *p*-cresol sulfate levels across different matrices remained unknown. Using targeted metabolomics, we systematically quantified the levels of unconjugated *p*-cresol and its main conjugate *p*-cresol sulfate across various peripheral matrices (urine, feces, plasma) and in brain samples collected in the same individuals. In fecal, urinary and plasma control samples, *p*-cresol was below the detection level but *p*-cresol became detectable in *p*-cresol-treated animals (Fig. 2B, C, D). *p*-Cresol was most abundant in urine in the low micromolar range, followed by feces and plasma in the nanomolar range. Compared to reference detection levels in untreated animals, *p-*cresol-treated mice exhibited a 1.58-fold, 9.33-fold, and 4-fold increase of *p-*cresol levels in feces, urine, and plasma, respectively (Fig. 2B, C, D, Supplementary Table 1). Initial measures in brainstem performed with the same Ultra Performance Liquid Chromatography coupled to tandem Mass Spectrometry (UPLC-MS/MS) method following derivatization by dansylation as for urine and plasma analysis did not allow detection of *p*-cresol in the brainstem, even in samples derived from *p*-cresol-treated animals (not shown). We therefore developed and validated a highly-sensitive method for quantifying *p*-cresol using derivatization with 1,2-dimethylimidazole-5-sulfonyl chloride (5-DMIS-Cl) and LC-MS/MS, which enhanced by 40-fold the sensitivity compared to traditional dansyl derivatization^34^. In brainstem samples collected in an independent cohort, we were able to detect *p*-cresol in the femtomolar range and to show that *p*-cresol-treated animals exhibited a 1.71-fold increase in *p*-cresol levels as compared to untreated animals (Fig. 2E, Supplementary Table 1).

*p*-Cresol sulfate, the major conjugate of *p*-cresol was detectable in all matrices, in both control and *p*-cresol-treated animals, within the micromolar range for feces, urine and plasma and within the picomolar range in the brainstem. *p*-Cresol-treated animals exhibited *p*-cresol sulfate increases of 1.81-fold, 10.11-fold, 2.27-fold and 1.34-fold in the feces, urine, plasma, and brainstem, respectively (Fig. 2B, C, D, E, Supplementary Table 1). Of note the fold increases in *p*-cresol sulfate following *p*-cresol-treatment were comparable to those observed for *p*-cresol levels. Moreover, the relative increases in *p-*cresol and *p-*cresol sulfate detected in our model fall within the range previously reported in urinary or fecal samples from ASD patients, which span a 1.26- to 3.33-fold increase (as summarized in Supplementary Table 2 and refs. ^20–30^). Compared to *p*-cresol levels, *p*-cresol sulfate levels were 11,833- to 13,440-fold higher in the feces and 544- to 801-fold higher in the urine, plasma, and brainstem (Supplementary Table 1). This confirms anterior studies showing that *p*-cresol is rapidly uptaken by the host and conjugated in *p*-cresol sulfate, which is its major circulating and central form^11,12^.

Then, to gain insights in the relationships between *p*-cresol and *p*-cresol sulfate levels across matrices, we performed Spearman’s rank-order correlation analyses on data collected in control and *p-*cresol-treated animals (Fig. 2F). Positive correlations were observed for plasmatic/urinary *p*-cresol and plasmatic/urinary/fecal *p*-cresol sulfate levels, showing that *p*-cresol treatment in drinking water leads to increases of *p*-cresol and *p*-cresol sulfate in all matrices studied.

### *p-*Cresol exposure reduces catecholamines and accelerates dopamine turnover specifically in the brainstem

**Fig. 3:**
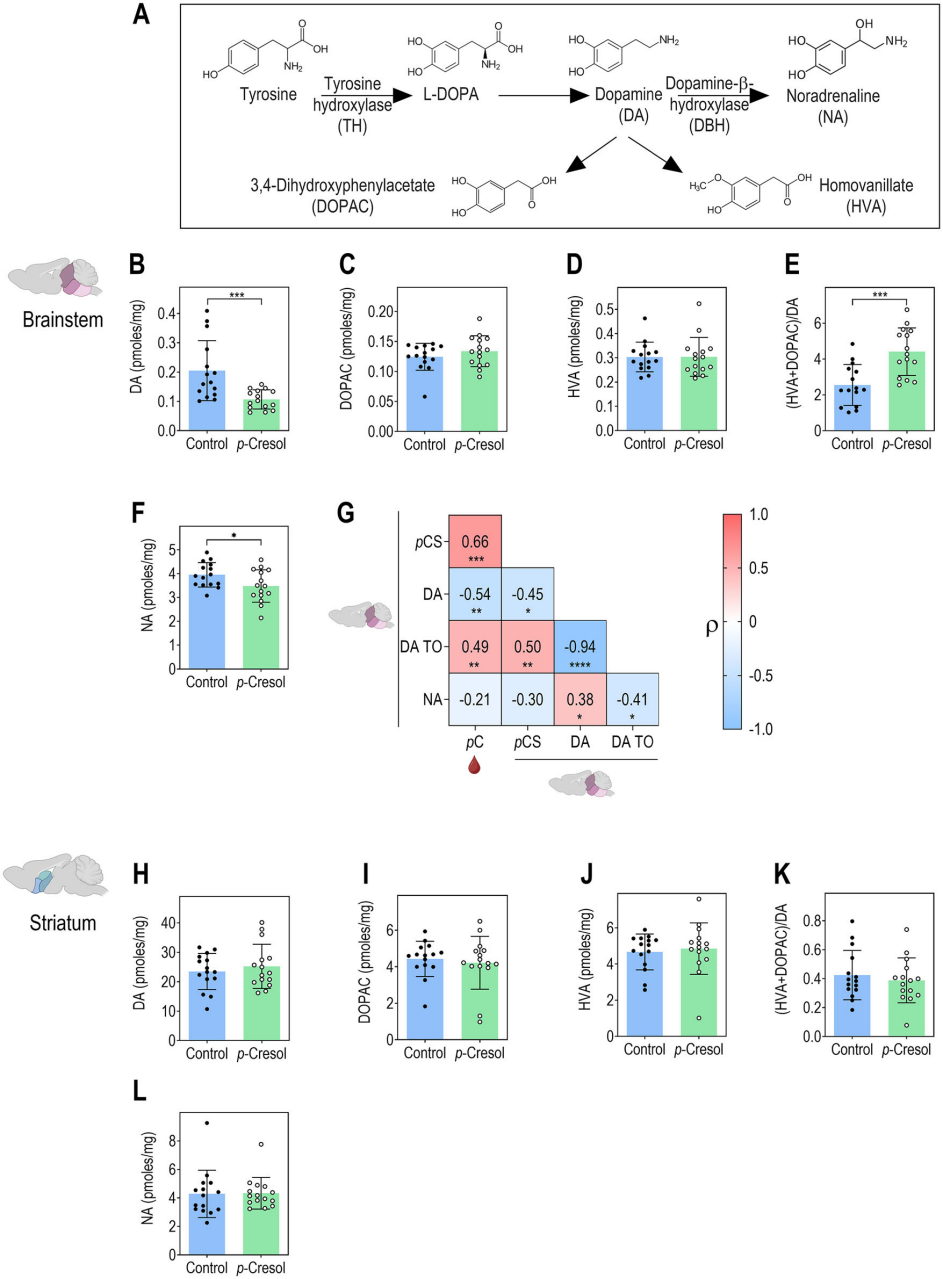
*p*-Cresol exposure reduces catecholamines and accelerates dopamine turnover in brainstem but not in striatum. **A** Catecholamines biosynthesis pathway. **B** Dopamine (DA) levels in brainstem. Unpaired *t* test: *t* = 3.531, df = 28, **p = 0.0015. **C** 3,4-Dihydroxyphenylacetic acid (DOPAC) levels in brainstem. Unpaired *t* test: *t* = 1.066, df = 28, *p* = 0.2955. **D** Homovanillic acid (HVA) levels in brainstem. Unpaired *t* test: *t* = 0.02409, df = 28, *p* = 0.9809. **E** DA turnover in brainstem. Unpaired *t* test: *t* = 4.106, df = 28, ***p = 0.0003. **F** Noradrenaline (NA) levels in brainstem. Unpaired *t* test: *t* = 2.129, df = 28: *p = 0.042. **G** Correlations between levels of *p*-cresol in plasma (red drop), *p*-cresol sulfate in brainstem, and catecholamines in brainstem (*n* = 30 (*n* = 15 Control, *n* = 15 *p*-Cresol) except for *p*-cresol levels in plasma, *n* = 28 (*n* = 14 Control, *n* = 14 *p*-Cresol)). Spearman’s ρ rank correlation coefficients test: *p < 0.05, **p < 0.01, ***p < 0.001, ****p < 0.0001. **H** DA levels in striatum. Unpaired *t* test: *t* = 0.7026, df = 28, *p* = 0.4881. **I** DOPAC levels in striatum. Mann-Whitney *U* test: *U* = 102, *p* = 0.682. **J** HVA levels in striatum. Unpaired *t* test: *t* = 0.4092, df = 28, *p* = 0.6855. **K** DA turnover in striatum. Unpaired *t* test: *t* = 0.6093, df=28, *p* = 0.5472. **L** NA levels in striatum. Unpaired *t* test: *t* = 0.06432, df = 28, *p* = 0.9492. **B**–**F**, **H**–**L** *n* = 15 Control, *n* = 15 *p*-Cresol. Data are presented as dot plots showing means ± standard deviation. **G** Heatmap of Spearman’s ρ rank correlation coefficients between variables pairs.

The presence of *p*-cresol in the brain, and its 1.71-fold increase in the brainstem of *p*-cresol-treated animals, suggested that *p*-cresol could exert direct effects in the brain. We previously reported that *p*-cresol-treated animals exhibited decreased excitability of VTA dopaminergic neurons, suggesting that *p*-cresol could impact catecholamine metabolism^36^. Both *p*-cresol and the main catecholamines DA and NA are derived from the essential amino acid tyrosine (Fig. 2A, Fig. 3A). Given that: (i) the brainstem contains the VTA, a critical dopaminergic region and the locus coeruleus (LC), the major central site for NA production (Fig. 1A), (ii) the striatum receives important dopaminergic inputs, we analyzed these regions in the same animals. In the brainstem, we found that *p*-cresol treatment decreased DA, while the levels of its main catabolites 3,4-dihydroxyphenylacetic acid (DOPAC) and homovanillic acid (HVA) were unaffected (Fig. 3B, C, D). Consequently, DA turnover was increased in *p*-cresol-treated animals compared to control (Fig. 3E). Additionally, we observed a slight, yet significant, decrease in NA in the brainstem of *p*-cresol treated mice (Fig. 3F). Further, correlation analyses revealed that the levels of plasma *p*-cresol and brainstem *p*-cresol sulfate negatively correlated with DA levels and positively correlated with DA turnover. In addition, DA levels were positively correlated to NA levels and negatively with DA turnover (Fig. 3G). Conversely, when examining striatal samples, no change in the levels of DA, DOPAC, HVA, DA turnover, or NA were observed, suggesting that *p*-cresol treatment effects were specific to the brainstem (Fig. 3H-L). Furthermore, in both the brainstem and the striatum, the levels of serotonin and its catabolite 5-hydroxyindoleacetic acid (5-HIAA) or serotonin turnover remain unchanged (Supplementary Fig. 1). These data collectively suggest that *p*-cresol spares the serotoninergic system and that it targets brainstem catecholamines.

### *p-*Cresol inhibits the activity of TH and DBH, key enzymes for catecholamine biosynthesis

**Fig. 4:**
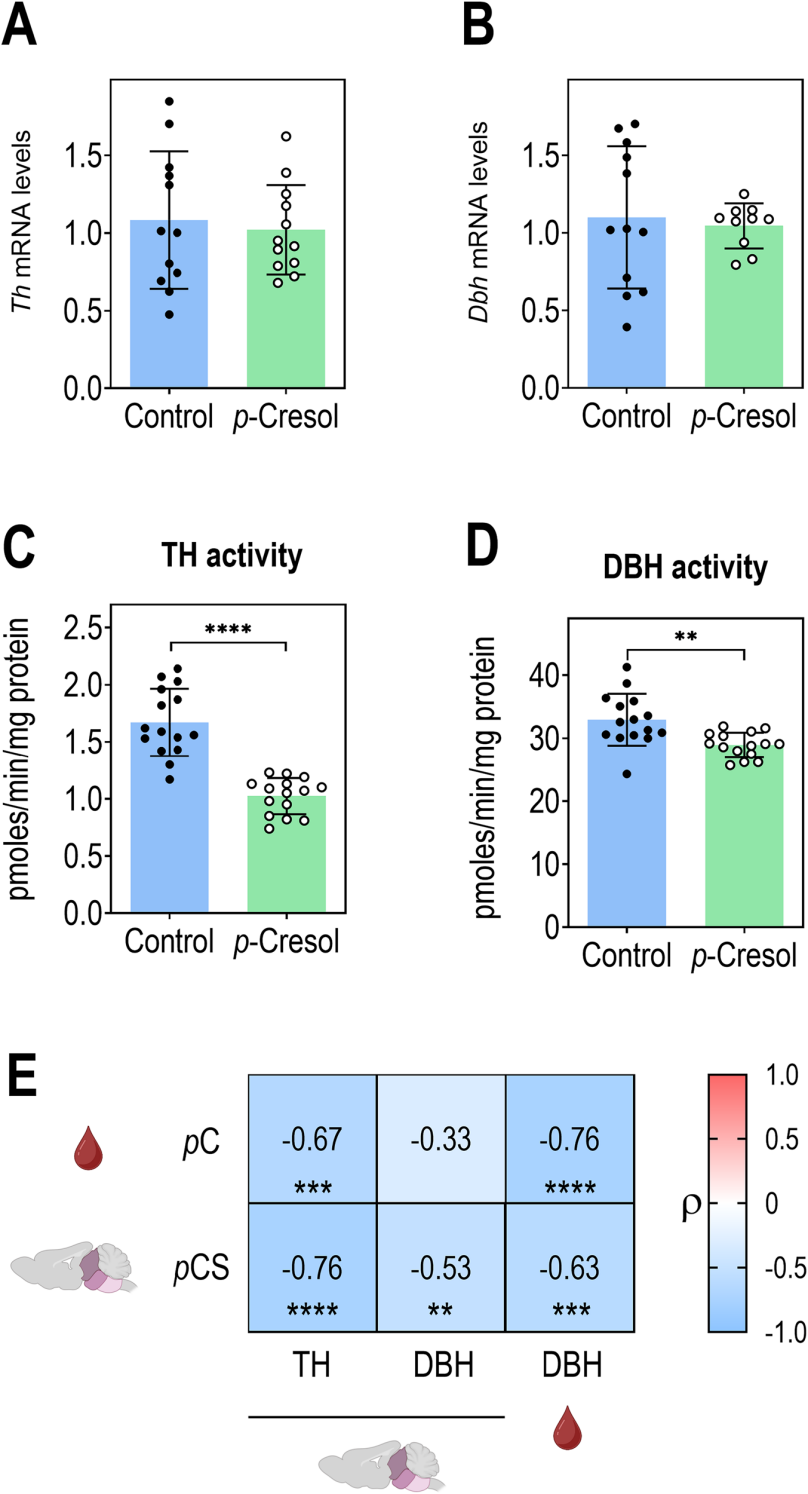
*p*-Cresol exposure reduces catecholamine-synthesizing enzymes activities. **A** Tyrosine hydroxylase (*Th*) mRNA levels in brainstem (*n* = 12 Control, *n* = 12 *p*-Cresol). Unpaired *t* test: *t* = 0.4119, df = 22, *p* = 0.6844. **B** Dopamine-β-hydroxylase (*Dbh*) mRNA levels in brainstem (*n* = 12 Control, *n* = 10 *p*-Cresol). Mann-Whitney *U* test: *U* = 59, *p* = 0.9742. **C** Tyrosine hydroxylase (TH) activity in brainstem (*n* = 15 Control, *n* = 15 *p*-Cresol). Unpaired *t* test: *t* = 7.468, df = 28, ****p < 0.0001. **D** Dopamine-β-hydroxylase (DBH) activity in brainstem (*n* = 15 Control, *n* = 15 *p*-Cresol). Unpaired *t* test: *t* = 3.397, df = 28, **p = 0.0021. **E** Correlations between *p*-cresol levels in plasma (red drop), *p*-cresol sulfate levels in brainstem, TH and DBH enzyme activity in brainstem and DBH enzyme activity in plasma (red drop) (*n* = 30 (*n* = 15 Control, *n* = 15 *p*-Cresol), except for *p*-cresol and *p*-cresol sulfate levels in plasma *n* = 28 (*n* = 14 Control, *n* = 14 *p*-Cresol)). Spearman’s ρ rank correlation coefficients test: **p < 0.01, ***p < 0.001, ****p < 0.0001. **A**–**D** Data are presented as dot plots showing means ± standard deviation. **E** Heat map of Spearman’s ρ rank correlation coefficients between variables pairs.

To further investigate whether *p-c*resol affects catecholamine synthesis, we first monitored the expression in the brainstem of the two rate-limiting enzymes of catecholamine synthesis: TH for DA production and DBH for NA production (Fig. 3A). *Th* and *Dbh* transcripts levels in the brainstem were unaffected by *p*-cresol treatment (Fig. 4A, B). However, when directly monitoring the activity of both enzymes in brainstem extracts, we observed that the activities of TH and to a lesser extent of DBH were reduced (Fig. 4C, D). The decrease in systemic DBH activity was also confirmed in the plasma of *p*-cresol-treated animals (Supplementary Fig. 2). Correlation analysis revealed that plasmatic *p*-cresol levels and brainstem *p*-cresol sulfate levels were negatively correlated with TH and or/DBH activity (Fig. 4E). These findings suggest that *p*-cresol could affect catecholamine biosynthesis by inhibiting both TH and DBH enzymes.

**Fig. 5:**
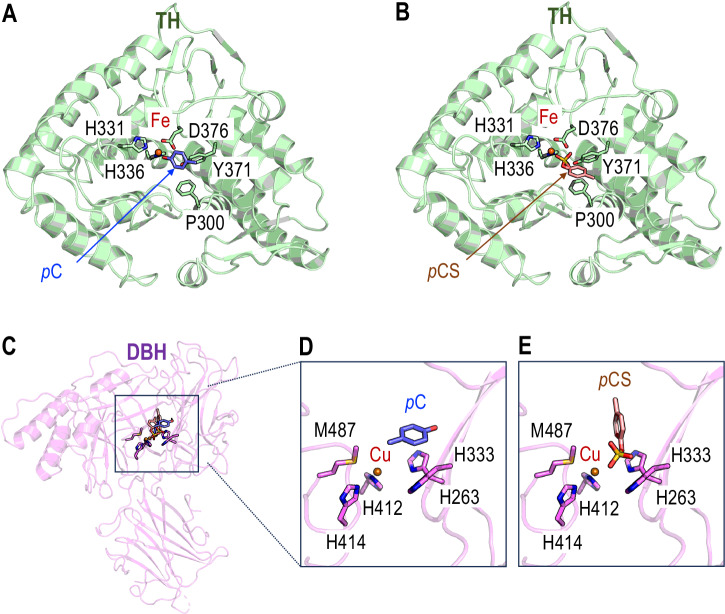
Optimal docking geometries of *p*-cresol and *p*-cresol sulfate within TH and DBH active sites. **A** Predicted docking geometry of *p*-cresol (*p*C, in blue) within the active site of tyrosine hydroxylase (TH, in green), showing interaction near the catalytic iron ion (Fe, in red). **B** Predicted docking geometry of *p-*cresol sulfate (*p*CS, in orange) within the TH active site (in green), positioned near the catalytic iron ion (Fe, in red). **C** Overview of docking geometries showing superimposed binding poses of *p*-cresol and *p-*cresol sulfate in complex with DBH (in purple). **D** Close-up view of the optimal binding pose of *p*-cresol (*p*C, in blue) within the DBH active site, showing proximity to the copper ion (Cu, in red). **E** Close-up view of the optimal binding pose of *p*-cresol sulfate (*p*CS, in orange) within the DBH active site, highlighting interaction with the catalytic copper ion (Cu, in red). All predictions were performed with HADDOCK.

Similar to tyrosine and DA, the respective substrates of TH and DBH enzymes, *p*-cresol and *p*-cresol sulfate exhibit a benzene aromatic ring with substituents in the para position (Figs. 2A, 3A, Supplementary Table 3). We therefore performed molecular docking simulations to test the direct binding of *p*-cresol and *p*-cresol sulfate on TH and DBH, based on their available crystal structures. Representations of the best docking geometries obtained with HADDOCK are shown in Fig. 5 and associated metrics for the top clusters of docking simulations for TH and DBH are provided in Supplementary Table 4 and Supplementary Table 5, respectively. For both metabolites and both enzymes, we identified binding poses within the catalytic pockets, near the Fe^2+^ ion in TH (Fig. 5A, B) and the Cu^2+^ ion in DBH (Fig. 5C, D, E). The catalytic sites of both enzymes contain aromatic amino acids, which can stabilize *p*-cresol through π-π stacking interactions. In TH, *p*-cresol is involved in T-shaped π-π stacking interactions with residues P300 and Y371 (Fig. 5A), ensuring its stability, and *p*-cresol sulfate is involved in a flat π-π stacking interaction with residue P300 (Fig. 5B). In DBH, *p*-cresol is involved in stacking interactions with H333 from the Cu_H_ binding site. Additionally, all of the geometries proposed by the top docking clusters exhibit an interaction between the hydroxyl radical of *p*-cresol or the sulfate of *p-*cresol sulfate with the metal cation. Docking simulations performed with the alternative software AutoDock Vina produced comparable geometries for the binding of *p*-cresol and *p*-cresol sulfate within the catalytic pockets and predicted interactions with the same amino acids within the active sites of both TH (Supplementary Fig. 3A, B), and DBH (Supplementary Fig. 3C, D, E).

To go further, we complemented the docking assays with AlphaFold3-based structural predictions, which allowed us to model the 3D structures of TH and DBH solely from their amino acid sequences, without relying on existing crystallographic data. This approach was particularly valuable for DBH, for the available crystal structure represents only a partial or conformationally specific state as reported in ref. ^37^. In AlphaFold3-predicted TH structures, both *p*-cresol or *p*-cresol sulfate engaged with π-π stacking interactions with P300 and interacted with the iron cofactor within the catalytic pocket of the enzymes, recapitulating the docking results (Supplementary Fig. 4A, B). Although, the AlphaFold2 model of DBH exhibited a global conformation that differed from the crystal structure used for docking, the binding geometries of *p*-cresol and *p*-cresol sulfate (Supplementary Fig. 4C, D, E) remained remarkably consistent with those derived from molecular docking (Fig. 5C, D, E). Together with the observed reduction of TH and DBH enzymatic activities, the in silico models support the notion that *p*-cresol and *p-*cresol sulfate can act as inhibitors by mimicking tyrosine or DA binding near the catalytic ion within the active sites of TH and DBH.

### *p-*Cresol treatment and DBH pharmacological blockade induce similar social deficits

**Fig. 6:**
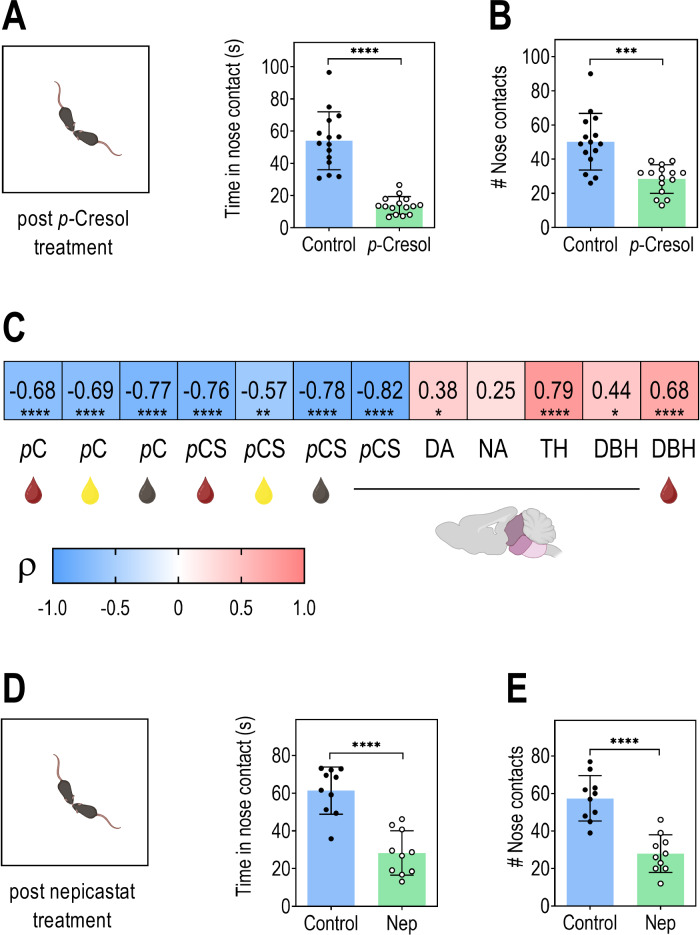
DBH inhibition by *p*-cresol or its pharmacological blockade induce social interaction deficits in mice. **A**, **B** Dyadic social interaction test in *p-*cresol treated mice (*n* = 15 Control, *n* = 15 *p-*Cresol). **A** Time spent in nose contact. Unpaired *t* test: *t* = 8.295, df = 28, ****p < 0.0001. **B** Number of nose contacts. Unpaired *t* test: *t* = 4.566, df = 28, ****p < 0.0001. **C** Correlations between social abilities measured with the time spent in nose contact, *p*-cresol (*p*C) levels in plasma, urine, feces, *p*-cresol sulfate (*p*CS) levels in plasma, urine, feces, brainstem, DA and NA levels in brainstem, TH and DBH enzyme activities in brainstem, and DBH enzyme activity in plasma (*n* = 30 (*n* = 15 Control, *n* = 15 *p-*Cresol*)* except for *p*-cresol and *p*-cresol sulfate levels in plasma *n* = 28 (*n* = 14 Control, *n* = 14 *p-*Cresol). Spearman’s ρ rank correlation coefficients test: *p < 0.05, **p < 0.01, ***p < 0.001, ****p < 0.0001. **D**–**E** Dyadic social interaction test 2 h post vehicle (Control) or nepicastat (Nep) treatment (*n* = 10 Vehicle, *n* = 10 Nep). **D** Time spent in nose contact. Unpaired *t* test: *t* = 6.090, df = 18, ****p < 0.0001. **E** Number of nose contacts. Unpaired *t* test: *t* = 5.903, df = 18, ****p < 0.0001. **A**, **B**, **D**, **E** Data are presented as dot plots showing means ± standard deviation. **C** Heatmap of Spearman’s ρ rank correlation coefficients between variables pairs.

To study the relationship between social abilities, *p*-cresol and *p*-cresol sulfate levels, catecholamine levels, TH and DBH activities, we scored dyadic social interactions in the same set of *p*-cresol-treated and control mice (Fig. 6A). As previously described^36^, *p*-cresol-treated mice exhibited social deficits, with a decrease in the total time spent in nose contacts and a reduction in the number of nose contact events (Fig. 6A, B). Further correlation analysis highlighted that plasmatic, urinary and fecal *p*-cresol and plasmatic, urinary, fecal and brainstem *p*-cresol sulfate levels exhibit negative correlations with social abilities assessed by the total time spent in nose contact (Fig. 6C). Also, brainstem DA, TH, and DBH activities correlated positively with social abilities, suggesting that catecholamine biosynthesis conditions adequate social behavior skills (Fig. 6C). To reinforce the mechanistic connection between direct inhibition of DBH by *p*-cresol and social behavior deficits observed in *p*-cresol-treated animals, we monitored the social behavior of wildtype mice acutely treated with nepicastat, a known and specific pharmacological inhibitor of DBH^38–40^. We found that, as compared to vehicle-treated control mice, nepicastat-treated mice exhibited a decrease in the total time spent in nose contacts and in the number of nose contact events (Fig. 6D, E). These findings show that DBH inhibition induces social behavior deficits in mice, mimicking the effects of *p*-cresol treatment.

## Discussion

Here, we investigated the biodistribution of *p*-cresol and its main host conjugate, *p*-cresol sulfate, in peripheral and central matrices. We also examined the impact of *p*-cresol on the biosynthesis of DA and NA in key brain catecholamine regions: the brainstem and striatum. In the brainstem, but not in the striatum, *p*-cresol decreased DA and NA levels, increased dopamine turnover, and reduced the activity of TH and DBH, two key enzymes in catecholamine biosynthesis. In silico docking simulations predicted that both *p*-cresol and *p*-cresol sulfate can bind within the catalytic pocket of both TH and DBH, acting as a competitive inhibitor for these enzymes. We also showed that pharmacological DBH blockade was sufficient to recapitulate the social deficits observed in *p*-cresol-treated mice. Together, our findings suggest that the direct inhibition of host central enzymes by *p*-cresol impairs catecholamine biosynthesis and contributes to social behavior deficits.

In this study, we show that *p*-cresol is a very low-abundance metabolite, while its conjugate *p*-cresol sulfate is easily detected and is over several hundred or thousand-fold more abundant than free *p*-cresol. In *p*-cresol-treated animals, *p*-cresol becomes detectable in all matrices, allowing the monitoring of its biodistribution. Both metabolites are most abundant in the feces and urine, followed by plasma, then brain. In the plasma, we report that, *p*-cresol circulates at low levels, within the nanomolar range, while *p*-cresol sulfate is in the micromolar range, with relative increases of 4-fold for *p*-cresol and 2.27-fold for *p-*cresol sulfate upon *p*-cresol treatment. Using a highly-sensitive LC/MS-MS method, we were able to detect minute amounts of *p*-cresol in the brain of control animals (in the femtomolar range) and to show that its levels were increased by 1.71-fold in the brain of *p*-cresol-treated animals. Conversely, *p*-cresol sulfate was found to be considerably more abundant in the brain (in the nanomolar range), showing a 1.34-fold increase. These effect sizes are with those reported in peripheral matrices of ASD patients (1.35 to 3.33-fold for urinary *p*-Cresol^23–28^, 1.26–2.58-fold for fecal *p*-Cresol^20–22^), thereby reinforcing the relevance of our model for investigating the pathophysiological effects of *p*-cresol. To date, circulating concentrations of *p*-cresol and *p*-cresol sulfate in the blood of ASD patients remain undetermined, preventing direct comparison with the levels measured in our model. Further clinical studies employing absolute quantification of circulating and excreted *p-*cresol and its conjugates, particularly in relation to ASD symptom severity and social deficits, are needed to clarify their potential role in the disorder.

Our data also suggest that *p*-cresol produced in the intestinal lumen by the gut microbiota passes the intestinal epithelium to enter the systemic circulation, crosses the blood-brain-barrier and reaches the brain parenchyma. *p-*Cresol is slightly amphiphilic as it contains a hydrophobic methyl group, a large non-polar aromatic ring and a polar hydroxyl group. This amphiphilic nature and its small size could facilitate its passive diffusion across lipid bilayers, including those of intestinal epithelial cells. Although specific active transport mechanisms for *p-*cresol have not been studied, its structural resemblance to tyrosine, a known substrate of the aromatic amino acid transporter SLC16A10, suggests potential interaction, which may similarly facilitate *p*-cresol uptake or excretion^41^. In contrast, *p-*cresol sulfate is a highly polar and hydrophilic metabolite, mainly generated in the liver via sulfation of *p*-cresol. Due to its high polarity, *p-*cresol sulfate is unlikely to cross lipid membranes by passive diffusion and instead relies on active transport mechanisms for tissue distribution and renal excretion^42^. Since we have detected it in the brain and shown its increase in *p*-cresol-treated animals, we can envision that either peripheral *p*-cresol sulfate reaches the brain via transporters, possibly via the organic anion transporters OAT1 or OAT3 (the latter also expressed in the brain)^43^. Alternatively, *p*-cresol sulfate could be formed in the brain from *p*-cresol, as described for a parent molecule 4-ethylphenol sulfate which was shown to be also produced by 4-ethylphenol sulfation in the brain by sulfotransferase A1^44^. Interestingly, it has been shown that ASD patients have global impairment of sulfation metabolism as shown by decreases phenol sulfotransferase activities, when compared to unaffected siblings or neurotypical individuals^45,46^. This deficit can lead to an overall decrease in *p*-cresol sulfo-conjugation, which could result in *p*-cresol brain accumulation.

Given that a fraction of *p*-cresol remains free in brain tissue and the higher abundance of its conjugate *p*-cresol sulfate in the brain, both molecules are positioned to exert direct effects on host central enzymes or receptors. Regarding their possible action in the brain, we provide in vivo and in silico evidence that the rate-limiting enzyme for catecholamine biosynthesis TH and DBH are direct molecular targets of the microbial metabolite *p*-cresol, but also its host-derived conjugate, *p*-cresol sulfate. The moderate, but consistent, decrease in DBH activity in brainstem extracts from *p*-cresol-treated animals align with prior in vitro studies demonstrating that *p*-cresol acts as a competitive inhibitor of DBH, mimicking DA binding^47,48^. Here, we extend these findings by showing that *p*-cresol sulfate, a more polar molecule and conjugate of *p-*cresol, yet highly abundant metabolite, may similarly contribute to DBH enzyme inhibition in vivo. Importantly, our data suggest that *p*-cresol and *p*-cresol sulfate more broadly affect catecholamine biosynthesis by inhibiting its rate-limiting enzyme TH, as shown by the marked reduction in TH activity and DA production we observed in the brainstem of *p*-cresol-treated animals. These changes mirror prior reports of decreased platelet catecholamines in children with ASD^49^, suggesting that microbial metabolite interference with catecholamine biosynthesis may represent a mechanistic link between gut dysbiosis and neurobehavioral phenotypes.

Crucially, our in silico models reveal a possible mechanism of action for *p*-cresol and *p*-cresol sulfate which share structural features with tyrosine (TH substrate) and DA (DBH substrate), including an aromatic ring with *para*-substituents. Our simulations predicted that both metabolites can bind within the catalytic pockets of TH and DBH, interacting directly with their metal cofactors (Fe²⁺ for TH and Cu²⁺ for DBH), and forming stabilizing π-π stacking interactions with conserved aromatic residues (e.g., P300 and Y371 in TH, H333 in DBH). Notably, *p-*cresol sulfate, despite its polarity, retains the reactive benzylic moiety, enabling similar binding geometries and interaction energies as *p*-cresol. Together, these biochemical and computational data provide compelling evidence that *p*-cresol and *p-*cresol sulfate can directly bind within TH and DBH catalytic cores, mimicking endogenous substrates and therefore potentially acting as competitive inhibitors. Importantly, our study suggests direct molecular interactions in vivo between microbial metabolites and central host enzymes as a mechanism mediating microbiota-brain communication.

Our data also suggest that the brainstem catecholaminergic nuclei, including the LC, VTA, and substantia nigra pars compacta (SNc), may be particularly susceptible to the toxic or inhibitory effects of microbial metabolites (e.g., *p-*cresol) or co-metabolites (e.g., *p*-cresol sulfate). First, compared to more internal or downstream projection areas like the striatum, the brainstem nuclei are anatomically more exposed to peripheral *p*-cresol passage because the commissural and dorsomedial subnuclei of the nucleus tractus solitarius (NTS), a part of the brainstem, appear more permissive to toxin influx as the BBB remains fenestrated in this area^50^. Also, the NTS contains A2 NA neurons that are consistently activated by treatments or situations that present actual or anticipated threats to bodily homeostasis, such as toxin exposure^50^. Both the LC and VTA are located adjacent to the fourth ventricle and dorsal brainstem surfaces, placing them in close contact with cerebrospinal fluid (CSF). This proximity increases the likelihood of direct brainstem exposure to CSF-borne toxins or microbial metabolites, such as gut-derived phenolic compounds like *p*-cresol and its sulfate conjugate^51^. This is in contrast to more internal striatal regions that are not directly bathed by CSF, which may reduce their vulnerability, possibly explaining the lack of effect of *p-*cresol observed in the striatum. Second, the brainstem LC, VTA and SNc are biochemically highly specialized, as they exhibit high expression levels of TH—the rate-limiting enzyme in catecholamine synthesis—and, in the case of the LC, DBH, which converts DA into NA^52,53^. This makes them potential primary targets for microbial-derived compounds that interfere with catecholaminergic metabolism. In contrast, the striatum, while densely innervated by SNC and VTA dopaminergic terminals, does not itself express high levels of TH or DBH or synthesize DA and is therefore less likely to be affected by toxins that target catecholamine-producing enzymes^54^. Third, SNc and VTA neurons are highly metabolically active, with long, highly branched axons and autonomous pacemaking activity driven by L-type calcium channels^55,56^. This results in sustained calcium influx and substantial oxidative stress, making them especially prone to mitochondrial dysfunction^55,56^. Inhibition of TH or DBH activity by microbial metabolites may further compromise their function by disrupting DA and NA synthesis, thereby priming toward dysfunction^54^. Altogether, the unique anatomical access to circulating metabolites, the critical reliance on catecholaminergic enzyme activity, and the intrinsic metabolic vulnerability of LC, VTA, and SNc neurons make brainstem catecholaminergic nuclei more likely to be affected by gut-derived microbial metabolites such as *p-*cresol and *p*-cresol sulfate, particularly when compared to the striatum.

Our findings support the notion that brainstem catecholamine disruption, via inhibition of TH and DBH can lead to social deficits enzymes in the brainstem can impair social behavior, even in the absence of detectable changes in striatal catecholamine levels. Brainstem DA levels and TH/DBH activities correlated positively with social abilities, supporting the widespread influence of dopaminergic and noradrenergic neurons projecting to key extra-striatal regions involved in social cognition and motivation. The LC, the brain’s primary source of NA, innervates the prefrontal cortex, amygdala, and hippocampus, regions critical for social behavior regulation^57^. Similarly, VTA dopaminergic neurons project to the prefrontal cortex and nucleus accumbens, where they regulate reward, motivation, and social engagement^58,59^. Inhibition of TH in these neurons could reduce DA release in projection targets and impairs behaviors related to social exploration^58,60^. While the striatum receives relatively sparse noradrenergic input^61^, its function is modulated indirectly by corticolimbic circuits influenced by NA and DA signaling. Thus, perturbing TH or DBH activity in the brainstem can disrupt the broader neuromodulatory networks that support adaptive social behavior, even without directly altering striatal catecholamine levels. This differential effect on DA and TH, NA and DBH aligns with our observed neurophysiological changes, including reduced VTA DA neuron excitability and selective impairments in social behavior (ref. ^36^ and this study). Disruption of NA synthesis, such as through DBH pharmacological inhibition or knockout in rodents, was previously shown to impair social memory and affiliative behavior^38,62,63^. We show here that pharmacological inhibition of DBH using nepicastat induced social deficits which mirrored the social impairments observed in *p*-cresol-treated mice. Of note, this effect on social behavior is unlikely to result from a reduced exploratory drive under DBH inhibition by nepicastat, as prior work has shown that the dose used here does not affect exploratory activity in the openfield^40^. Altogether, these findings provide new evidence that DBH inhibition by *p*-cresol and *p*-cresol sulfate could contribute to social deficits and generally highlight the importance of microbial metabolites in modulating social behaviors.

Our study has some limitations that should be acknowledged. First, we used only male mice of the C57Bl/6J strain, which limits the ability to assess sex-specific effects of *p-*cresol and may constrain the generalizability of the results. Second, our post-weaning exposure paradigm, based on direct *p*-cresol administration via drinking water, does not fully recapitulate the complexity of microbiota-derived *p*-cresol production from early development through adulthood. Third, while we focused on the striatum and midbrain, investigating catecholamine biosynthetic pathways in more specific brain subregions involved in social reward, such as the VTA, prefrontal cortex or nucleus accumbens, could provide more precise insights into the extent of catecholamine dysfunction caused by *p*-cresol-mediated inhibition of TH and DBH, and how these alterations relate to social behavioral deficits. Finally, future pre/probiotic-based dietary or pharmacological interventions aimed at reducing *p*-cresol levels or restoring catecholaminergic function would help validate the pathophysiological relevance of the mechanisms identified in this study.

In conclusion, our study provides evidence that the microbial metabolite *p*-cresol and its host conjugate *p*-cresol sulfate directly impair catecholamine biosynthesis in the brainstem, through direct inhibition of the key enzymes TH and DBH. The convergent effects observed in mice treated with nepicastat, a selective DBH inhibitor, further underscores the critical role of DBH in modulating social behavior. These findings identify disrupted catecholamine biosynthesis *via* inhibition of host central enzymes as a mechanism by which microbial metabolites may contribute to social deficits in ASD. By uncovering direct molecular interactions between gut-derived compounds and host neurochemical pathways, our work opens new avenues for investigating how microbial metabolites influence brain function, highlighting the need to systematically map their molecular targets, including enzymes and receptors, within brain circuits governing social processes.

## Methods

Supplementary Methods are available in Supplementary Information.

### Animals

#### *p*-cresol treatment

Weaned C57BL/6J male mice (21–28 day-old) were obtained from Charles River (France). Only males were considered in this study, as the ASD sex ratio is biased towards 3 males diagnosed for 1 female^64^. Upon arrival, animals were randomly allocated to the experimental groups and housed in cages of 4 to 5 animals. Cages were medium-size open cages filled with wooden bedding, plastic house and enrichment with nesting material (cotton pads), in a temperature (22–24 °C) and hygrometry (70–80%)-controlled room with a 12 h light/dark cycle (lights on from 7:00 a.m. to 7:00 p.m.) with *ad libitum* access to water and food (standard chow, reference 4RF25, Mucedola). Treatment with *p-*cresol (reference W233706-SAMPLE-K, Sigma-Aldrich) was dispensed in sterile drinking water at a concentration of 0.25 g/L (Fig. 1A). Based on a mean body mass of 25 g and a mean drinking water consumption of 5 mL/24 h, this is equivalent to a dose of 50 mg/Kg/24 h *per os*. We have previously described that this treatment was not interfering with basic physiological parameters (drink/food consumption, body weight) but led to social interaction deficits, with no impact on locomotor activity, exploratory behavior, anxiety or cognition^36^.

#### Nepicastat treatment

C57BL/6 J male mice (9-weeks old) obtained from Charles River (France) were acutely treated with Nepicastat hydrochloride (Biotechne #5037, France) intraperitoneally (i.p.) at a dose of 50 mg/kg, a previously reported dose effective to inhibit DBH^38–40^, without altering locomotor activity or exploratory behavior in the openfield^40^. Nepicastat was dissolved at 10 mg/mL in a sterile vehicle solution containing 0.29% DMSO and 0.9% NaCl and injected at 5 µL/g mouse.

#### Dyadic social interactions

After a 4-week *p-*cresol treatment or 2 h post-acute nepicastat treatment, direct social interactions were recorded in an open-field arena with low light intensity (15 Lux). The subject mouse was placed in presence with an unfamiliar sex- and age-matched interactor, and their interactions recorded for 10 min. Each recording session included four individual animals—two controls (or vehicle-treated) and two treated—randomly assigned to the four simultaneously monitored open fields. Manual scoring by an experienced experimenter blind to the experimental group was performed *a posteriori* by recording the number of nose contacts and time spent in nose contact^36^.

#### Samples collection

Urine and fecal pellets were collected in Eppendorf tubes immediately after excretion. Prior to blood and brain sampling, mice were euthanized by lethal intraperitoneal injection of sodium pentobarbital (250 mg/kg of body weight). Blood (0.9 ml) was collected by cardiac puncture using a syringe pre-filled with 100 μl of citrate buffer (45 mM sodium citrate, 25 mM citric acid, pH 4.5) and then centrifuged (10,000 × *g*, 2 min., 4 °C) to collect plasma. Brain regions were dissected as depicted in Fig. 1B. The brain was placed in an ice-cold coronal mouse brain matrix (RBMS 200C, 1 mm, World Precision Instruments) and dissected as described in Supplementary Methods. A razor blade was positioned rostral to the midbrain (Bregma -3 mm) and one at the cervico-medullary junction (Bregma -8 mm), and the segment was extracted. Using fine scissors, the brainstem (comprising the medulla, the pons, and the midbrain incl. VTA and substantia nigra) was separated from the overlying cerebellum. The striatum (comprising the ventral striatum, the basal forebrain and the caudate putamen) was finely dissected out from the sections lying between Bregma +1.70 mm → −0.46 mm. All samples were snap-frozen in liquid nitrogen, and stored at −80 °C until use.

### Targeted metabolomics for determination of *p*-cresol in biological matrices

#### Determination of p-cresol in feces, urine, and plasma

Thawed fecal pellets were extracted as previously described^36,65^. Plasma samples were deproteinized using Sirocco™ plasma protein filtering plates (Waters, USA). Thawed urine samples were centrifuged at 1200 × *g* for 10 min prior analysis. Fecal, urinary or plasmatic supernatants were spiked with an internal standard (*p*-cresol-d8 1 ng/mL, Eurisotop, France). Samples were subjected to dansyl chloride-derivatization to improve signal intensity of low-abundance phenolic compounds in UPLC-MS/MS analysis, as described in refs. ^66,67^. Samples were then analyzed using a Waters ACQUITY UPLC system with a binary solvent delivery manager and sample manager and coupled to a tandem quadrupole-time-of-flight (Q-TOF) mass spectrometer equipped with an electrospray interface (Waters, USA) as described in Supplementary Methods and ref. ^36^.

#### Determination of *p*-cresol in brain

To reach ultra-high sensitivity, 5-DMIS-Cl-derivatization was used instead of dansylation, as described in ref. ^34^. Samples were analyzed using a Waters Acquity UPLC system (Waters, USA) coupled to a Xevo TQ-S Micro triple quadrupole mass spectrometer, equipped with an electrospray interface (Waters, USA) as described in Supplementary Methods and ref. ^34^.

### Targeted metabolomics for determination of *p*-cresol sulfate in biological matrices

Samples were pre-processed as for *p*-cresol determination according to refs. ^36,68^. Samples were analyzed using a Waters Acquity™ UPLC HSS T3 1.8 μm column connected with an Acquity™ UPLC HSS T3 1.8 μm VanGuard pre-column (Waters, USA) as described in Supplementary Methods and ref. ^68^.

### Quantification of catecholamines (DA, NA) and their catabolites in brain tissue

The quantities of catecholamines (DA and NA) and the main DA catabolites DOPAC and HVA were measured by electrochemical detection on a serial array of four flow-through graphite coulometric electrodes (Ultimate 3000, Thermo Fisher Scientific), as described in Supplementary Methods.

### Quantification of serotonin and 5-HIAA in brain tissue

The quantities of serotonin and its main metabolite 5-HIAA were measured fluorometric detection on a Vanquish UPLC system (Thermo Fisher Scientific, France), adapted from ref. ^69^ and as described in Supplementary Methods.

### Quantitative RT-PCR

Total RNA was extracted from brainstem tissue using the RNeasy kit (Qiagen, Germany) and reverse transcription (RT) reaction was performed using the Superscript II RT-PCR system (Invitrogen, USA). Real-time PCR reactions were carried out using the Syber Green I qPCR core Kit (Eurogentec, Belgium) in a LightCycler system (Roche, USA), using primer pairs for *Th*, *Dbh*, and TATA Box Binding Protein (*Tbp*) mRNAs, as described in the Supplementary Methods. The 2^-∆∆Ct^ method^70^ was used to analyze the relative changes in *Th* and *Dbh* mRNAs relative to the *Tbp* mRNA used as reference.

### TH and DBH activity assay

TH enzymatic activity was determined by radioenzymology, based upon the release of ^3^H_2_O from ^3^H-[3,5]-L-tyrosine, according to Reinhard et al.^71^. DBH enzymatic activity was measured by converting (2-^14^C) tyramine into (2-^14^C) octopamine, which is then subjected to periodate cleavage to form (^14^C) *p-*hydroxybenzaldehyde, according to Bouclier et al. and Cressant et al.^72,73^. Both assays are detailed in Supplementary Methods.

### Molecular docking of *p-*cresol and *p*-cresol sulfate to TH and DBH

Molecular docking assays were performed using the HADDOCK web server based on solved protein structures of TH from *Rattus norvegicus* and human DBH (PDB identifiers: 1TOH and 4ZEL, respectively)^37,74,75^, as detailed in the Supplementary Methods. Ligand structures were generated from SMILES code using the ELBOW tool from the Phenix suite^76,77^. To cross-validate the results obtained with HADDOCK, molecular docking was also performed using the AutoDock Vina software via the SwissDock web server^78,79^.

### Structural prediction of *p-*cresol and *p*-cresol sulfate binding to TH and DBH

The structures of TH (amino-acid sequence PDB identifier: 1TOH) and DBH (using the full-length human amino-acid sequence (UniProt identifier: P09172)) in complex with *p-*cresol and *p*-cresol sulfate were predicted using AlphaFold3^80^. Both the multiple sequence alignment and inference steps were performed using a local installation of AlphaFold.

### Statistics and reproducibility

Data were first tested for normality using the Kolmogorov-Smirnov test. When the assumption of normality was met, comparisons between two independent groups were performed using the two-tailed Student *t* test while the one-sample *t* test with the limit of detection (LOD) as a reference was used when values below the detection threshold were obtained in control groups. When normality was not met in either one of the groups or both, comparisons between two independent groups were performed using the two-tailed Mann–Whitney *U* test. For correlation analyses, pairwise associations between variables and their corresponding *p* values were assessed using Spearman’s rank ρ correlation coefficient. Statistical significance was defined as *p* < 0.05 for all tests. Plotting and statistical analyses were conducted using GraphPad Prism version 8.00 (GraphPad Software, USA). A full report of statistical analyses, including sample sizes, normality testing, statistical test selection, group comparisons (degree of freedom, test statistic values, and *p* values), and correlation details (ρ, ρ confidence intervals, and *p* values) are provided either in the figure legends and/or in Supplementary Data file 1, while raw data are provided in Supplementary Data file 2.

### Ethics statement for animal housing and experimentation

Animal housing and experimentation were conducted in facilities certified by regional authorities (Direction Départementale de Protection des Populations des Alpes-Maritimes, accreditation #C-06-152-5). The study was conducted in accordance with procedures approved by the local ethics committee for animal experimentation (Ciepal-Azur) and the Ministère de l’Enseignement Supérieur et de la Recherche (APAFIS), in agreement with the European Communities Council Directive (2010/63EU) for animal experiments (Agreements references: APAFIS #21355-2019062414391395 v3, APAFIS #51032-2024082612133352 v5).

### Reporting summary

Further information on research design is available in the Nature Portfolio Reporting Summary linked to this article.

## Supplementary information

Supplementary Information

Description of additional supplementary files

Supplementary Data 1

Supplementary Data 2

Reporting Summary

## Data Availability

Detailed report of statistics (normality of data, detailed statistics for group comparison and correlations) are provided in Supplementary Data 1. The datasets generated and analyzed during the current study are provided in Supplementary Data 2.
