# Supplementary material for: The autism-linked gut microbial metabolite p-cresol inhibits host catecholamine biosynthesizing enzymes to elicit social deficits
Source: Commun Biol. 2025 Nov 22;8:1800. doi: 10.1038/s42003-025-09207-0 (PMC12722727; doi:10.1038/s42003-025-09207-0)
Supplement: Supplementary file 1 — Supplementary Information [file 42003_2025_9207_MOESM1_ESM.pdf]

SUPPLEMENTARY MATERIAL TO:

The autism-linked gut microbial metabolite *p*-cresol inhibits host catecholamine biosynthesizing enzymes to disrupt their biosynthesis and elicit social deficits

Geoffroy Mallaret[1,#], Juliette Canaguier[1,#], Jacques Callebert[2,#], Nicolas Caramello[3], David Fabregat Safont[4, 5], Nicolas Glaichenhaus[1, 6], Oscar J. Pozo[4], Jean-Marie Launay[2, 6], Laetitia Davidovic[1, 6]*

[1]Université Côte d'Azur, CNRS UMR7275, INSERM U1318, Institut de Pharmacologie Moléculaire et Cellulaire, Valbonne, France.

[2]Service de Biochimie et Biologie Moléculaire, INSERM U942, Hôpital Lariboisière, AP-HP, Paris, France.

[3] Univ. Grenoble Alpes, CNRS, CEA, Institut de Biologie Structurale (IBS), 71 Avenue des Martyrs, CS 10090, 38044 Grenoble Cedex 9, France.

[4]Applied Metabolomics Research Group, Hospital del Mar Research Institute, Barcelona, Spain.

[5]Environmental and Public Health Analytical Chemistry, Research Institute for Pesticides and Water (IUPA), Univ. Jaume I, Castelló, Spain.

[6]Alliance FondaMental, Créteil, France.

SUPPLEMENTARY METHODS

SUPPLEMENTARY TABLES: 5

SUPPLEMENTARY FIGURES: 4

SUPPLEMENTARY METHODS

Ethics statement for animal housing and experimentation

Animal housing and experimentation were conducted in facilities certified by regional authorities (Direction Départementale de Protection des Populations des Alpes-Maritimes, accreditation #C-06-152-5). The study was conducted in accordance with procedures approved by the local ethics committee for animal experimentation (Ciepal-Azur) and the Ministère de l'Enseignement Supérieur et de la Recherche (APAFIS), in agreement with the European Communities Council Directive (2010/63EU) for animal experiments (Agreements references: APAFIS #21355-2019062414391395 v3, APAFIS #51032-2024082612133352 v5).

Animals

p-Cresol treatment. Weaned C57BL/6J (authenticated Jax® mouse strain) male mice (21 to 28 days old) were obtained from Charles River (France). Only males were considered in this study, as the ASD sex ratio is biased towards 3 males diagnosed for 1 female [1]. Upon arrival, animals were randomly allocated to the experimental groups and housed in cages of 4 to 5 animals. Cages were medium-size open cages filled with wooden bedding, plastic house and enrichment with nesting material (cotton pads), in a temperature (22–24 °C) and hygrometry (70–80%)-controlled room with a 12 h light/dark cycle (lights on from 7:00 a.m. to 7:00 p.m.) with *ad libitum* access to water and food (standard chow, reference 4RF25, Mucedola). Mice were habituated for 6 days in our animal facility prior to treatment with *p*-cresol (reference W233706-SAMPLE-K, Sigma-Aldrich) dispensed in sterile drinking water at a concentration of 0.25 g/L (Figure 1A). Bottles were renewed twice a week. Based on a mean body mass of 25 g and a mean drinking water consumption of 5 mL/24 h, this is equivalent to a dose of 50 mg/Kg/24 h *per os*. We have previously described that this treatment was not interfering with basic physiological parameters (drink/food consumption, body weight) but led to social interaction deficits, with no impact on locomotor activity, exploratory behavior, anxiety or cognition [2].

Nepicastat treatment. C57BL/6J male mice (9-weeks old) obtained from Charles River (France) were habituated for 6 days to our animal facility. Mice were then acutely treated with Nepicastat hydrochloride (Biotechne #5037) intraperitoneally (i.p.) at a dose of 50mg/kg, a previously reported dose effective to inhibit DBH [3–5]. Nepicastat was dissolved at 10mg/mL in a sterile solution containing 0.29% DMSO and 0.9% NaCl and injected at 5µL/g mouse.

Dyadic social interactions. After a 4-week *p*-cresol treatment or 2 h post-acute nepicastat treatment, direct social interactions were recorded in an open-field arena with low light intensity (15 Lux). The subject mouse was placed in presence with an unfamiliar sex- and age-matched interactor, and their interactions recorded for 10 min. Each recording session included four individual animals—two controls (or vehicle-treated) and two treated—randomly assigned to the four simultaneously monitored open fields. Manual scoring by an experienced experimenter blind to the experimental group was performed *a posteriori* by recording the number of nose contacts and time spent in nose contact [2].

Samples collection. For urine and feces samples collection, mice were individually placed in clean empty plastic cages. Urine and fecal pellets were collected in Eppendorf tubes immediately after excretion with a sterile pipette or tweezers, respectively, to avoid cross-contamination. Samples were snap-frozen in liquid nitrogen and stored at -80°C until analysis. Prior to blood and brain sampling, mice were euthanized by lethal intraperitoneal injection of sodium pentobarbital (250 mg/kg of body weight). The thoracic cage was opened and blood (0.9 ml) was collected by cardiac puncture using a syringe pre-filled with 100 µl of citrate buffer (45 mM sodium citrate, 25 mM citric acid, pH 4.5). The blood sample was transferred to an ice-cold, clean 1.5ml Eppendorf tube, inverted 3 times and kept on ice. Samples were then centrifuged (10,000 g, 2 min., 4°C), and plasma was collected in clean Eppendorf tubes that were immediately snap-frozen in liquid nitrogen and stored at -80°C until analysis. As depicted in Figure 1B, brain regions were dissected as follow. The brain was carefully removed from skull, the olfactory bulbs and hypothalamus were removed with fine tweezers, and the brain was placed in an ice-cold, stainless-steel, coronal mouse brain matrix (RBMS 200C, 1mm, World Precision Instruments). A razor blade was positioned rostral to the midbrain (Bregma -3 mm) and one at the cervico-medullary junction (Bregma -8 mm), and the segment was extracted.

Using fine scissors, the brainstem (comprising the medulla, the pons, and the midbrain incl. VTA and substantia nigra) was separated from the overlying cerebellum. The striatum (comprising the ventral striatum, the basal forebrain and the caudate putamen) was finely dissected out from the sections lying between Bregma +1.70 mm → -0.46 mm. Immediately after dissection, brain regions were transferred to an ice-cold clean Eppendorf tube, snap-frozen in liquid nitrogen, and stored at -80°C until use.

Targeted metabolomics for determination of *p*-cresol in biological matrices

Determination of p-cresol in feces, urine, and plasma. Thawed fecal pellets were extracted as previously described [2,6]. Briefly, fecal pellets were homogenized in ice-cold water (1:1 w:v ratio), vortexed 30 s, ultrasonicated for 2 min, then subjected to ultracentrifugation (175,000×g, 30 min, 4 °C). Plasma samples were pre-processed in order to remove proteins: the samples were thawed on ice and 40 µL of sample was added into a 96-well Sirocco™ plasma protein filtering plate (Waters) containing 180 µL of methanol: acetonitrile (1 : 1, v/v). The plates were vortexed for 5 min, afterwards kept at 4 °C for 10 min to promote more protein precipitation followed by 5 min at room temperature. A 96 × 1 mL plate for the UPLC autosampler (Waters) was placed underneath the protein filtering plate and vacuum was applied to the manifold. When the filtering plates were dry, 90 µL of methanol was added to each well to further extract compounds from the precipitated protein and vacuum was connected until dryness. This step was repeated once. The solvent was evaporated by using a cooled vacuum centrifuge and the dry samples were re-dissolved in 200 µL in mobile phase A. Thawed urine samples were centrifuged at 1,200g for 10 min and 20 µl of the supernatant was 10× diluted in water containing 5% methanol (v/v) and the internal standards.

After adding an internal standard (1 ng/mL of *p*-cresol-d8, Eurisotop, St-Aubin, France) to fecal, urinary or plasmatic supernatants, prior to Liquid Chromatography coupled to tandem Mass Spectrometry (LC-MS/MS) analysis, we performed a derivatization step with dansyl chloride as described in Korytowska et al., 2019 [7]. Dansylation was shown to improve signal intensity in LC-MS/MS analysis of low-abundance phenolic compounds [8]. One hundred µL of supernatants were mixed with 300 µL of an acetonitrile solution vortexed, incubated at -20 °C for 20 min and centrifuged (12,000×g, 7 min, 4 °C). Two

hundred µL of the resulting supernatant were mixed with 50 µL of 0.1 M carbonate-bicarbonate solution (pH = 10), 125 µL of water, and 125 µL of dansyl chloride at a final concentration of 0.5 mg/mL. After vortexing, the mixture was incubated for 10 min at 60 °C and extracted with 2.5 mL of hexane. Hexane residues were air-dried and reconstituted with 1 mL of acetonitrile-water (1:1, v/v). Fifteen microliters of sample were injected and analyzed using a Waters ACQUITY ultraperformance liquid chromatography (UPLC) system equipped with a binary solvent delivery manager and sample manager (Waters Corporation, Milford, MA, USA) and coupled to a tandem quadrupole-time-of-flight (Q-TOF) mass spectrometer equipped with an electrospray interface (Waters Corporation). Quantifications were performed by referencing calibration curves obtained with internal standards. Unconjugated *p-c*resol was then identified by comparison with the exact mass and retention time of the reference standards. Blank and pooled quality control (QC) samples in several dilutions were included in the analysis to ensure that the measurements were reproducible. There were no external contaminations and the monitored analyte were responding linearly to the detector.

Determination of p-cresol in brain. To reach ultra-high sensitivity, 1,2-dimethylimidazole-5-sulfonyl chloride (5-DMIS-Cl)-derivatization was used instead of dansylation, as described in our previous study [9]. Briefly, 25 mg of brainstem sample was accurately weighted in a previously cleaned glass tube. Then, 20 µL of internal standard (*p*-cresol-d3) solution at 100 ng/mL and 500 µL of ultrapure water were added, and sample was homogenized using an Ultra-Turrax T10 (IKA-Werke GmbH & Co, Staufen, Germany). For derivatization, 100 µL of the $NaHCO_3/Na_2CO_3$ buffer and 100 µL of a 5-DMIS-Cl solution at 3 mg/mL in acetone (daily prepared) were added to each tube, mixed for 5 s using a vortex, and incubated at 60 ºC for 15 min. After that, derivatized *p*-cresol was extracted using a liquid-liquid extraction (LLE) adding 1 mL of ultrapure water, 200 µL of a saturated NaCl solution, and 4 mL of ethyl acetate. Ethyl acetate was dried under nitrogen stream at < 40 ºC, and extracts were reconstituted in 100 µL of water:methanol 1:1 (v:v). 1 µL of extract was injected and analyzed using a Waters Acquity UPLC I-Class ultra-high performance liquid chromatography system (Waters Corporation, Milford, MA, USA) coupled to a Xevo TQ-S Micro triple quadrupole mass spectrometer (LC-MS/MS), equipped with an electrospray interface (Waters Corporation). Quantifications were

performed by external calibration using the ration between the analyte and the internal standard as response. *p*-Cresol was identified by chromatographic retention time and ion ratios between reference standards and authentic samples.

Targeted metabolomics for determination of *p*-cresol sulfate in biological matrices

Samples were pre-processed as for *p*-cresol determination (see above) and fecal, urinary, plasmatic, and brainstem supernatants was diluted in mobile phase A spiked with the internal standard prior to analysis [10]. The chromatographic separation was performed on an Acquity™ UPLC System using a Waters Acquity™ UPLC HSS T3 1.8 μm 2.1 × 100 mm column connected with an Acquity™ UPLC HSS T3 1.8 μm VanGuard pre-column 2.1 × 5 mm (Waters, Milford, USA). 0.075% (v/v) formic acid in milliQ water (Millipore) as solvent A and MeOH as solvent B were mobile phases used for the gradient elution. 50% acetonitrile in 0.1% formic acid (v/v) was used as strong needle wash solvent; 10% acetonitrile (v/v) in milliQ water as seal wash solvent and 10% acetonitrile in 0.1% formic acid (v/v) as weak needle wash solvent. The injection volume was 7.5 μL (partial loop with needle overfill). The gradient steps ran at a column temperature of 40 °C is as follows: time (min.) 0/flow (mL min^{-1}) 0.55/A:B 98:2; 1/0.55/98:2; 1.5/0.43/75:25; 1.9/0.43/50:50; 4.1/0.6/20:80; 4.5/0.6/0:100; 4.9/0.6/0:100; 5.1/0.55/98:2; 6/0.55/98:2. MSMS parameters were optimized for single analytes by individual direct infusions of MeOH solutions with a compound concentration of approximately 10 μg mL^{-1} and an infusion flow rate of 5–10 μL min^{-1}. The target analyte was detected in MRM negative electrospray ionization mode using argon collision gas. The capillary voltage was set to 3.50 kV, source temperature to 150 °C, desolvation temperature to 350 °C, desolvation gas flow to 900 L h−1 and cone gas flow to 50 L h^{-1}. The primary transition was used for the quantification and the secondary for the confirmation of the compound identity. Peak integration was performed in QuanLynx Application Manager included with MassLynx Software 4.1 by Waters.

Quantification of catecholamines (DA, NA) and their main metabolites (DOPAC, HVA) in brain tissue

The levels of DA, NA and their respective metabolites were quantified using Ultra Performance Liquid Chromatography (UPLC). Prior to analysis, brainstem or striatum

samples were crushed in 0.2 M perchloric acid, containing ascorbic acid (0.2 µM) and EDTA (0.2 µM), and centrifuged after sonication at 9,000 g for 15 min at 4°C. The supernatants were collected and passed through a 10000 MWCO (Da) filter (Amicon) by centrifugation at 9,000 g for 15 min at 4°C. The quantities of catecholamines (DA and NA) and their main metabolites (DOPAC and HVA for DA) were then measured in a 50 µL aliquot of the ultrafiltrate by electrochemical detection on a serial array of four flow-through graphite coulometric electrodes (Ultimate 3000, Thermo Fisher Scientific). Results were expressed as pmoles/mg of tissue. Analysis, data reduction and peak identification were fully automated.

Quantification of serotonin and 5-hydroxyindoleacetic acid in brain tissue

The quantities of serotonin and its main metabolite 5-hydroxyindolacetic acid (5-HIAA) were then measured in another 50 µL aliquot of the ultrafiltrate by fluorometric detection on a Vanquish UPLC system (Vanquish, Thermo Fisher Scientific), adapted from Kema et al.,1993 [11]. Results were expressed as pmoles/mg of brain tissue. Analysis, data reduction and peak identification were fully automated.

Quantitative RT-PCR

Total RNA was extracted from brainstem tissue using the RNeasy kit (Qiagen, Hilden, Germany) and digested with DNAase I (Turbo DNAse, Ambion), according to the manufacturers' protocol. RNA integrity was verified on a 1% agarose gel. Reverse transcription (RT) reaction was performed on 250 ng of RNA, using the Superscript II RT-PCR system (Invitrogen, Carlsbad, California, USA) according to the manufacturers' protocol. Real-time PCR reactions were carried out using the Syber Green I qPCR core Kit (Eurogentec, Liège, Belgium) in a LightCycler system (Roche, USA). The qPCR program comprised the following steps: 1. Initial Denaturation / Enzyme Activation (95°C, 5 min), 2. Denaturation (95°C,15 s), 3. Annealing/Extension (60°C, 20 s), repeated 40 times, 4. Melting Curve (65°C → 95°C, 0.1°C/sec ramp. The comparative threshold cycle (Ct) for the amplicons of each sample was determined by the LightCycler software and normalised to the corresponding Ct of TATA Box Binding Protein (*Tbp*)

mRNA used as a reference gene. Finally, the $2^{-\Delta\Delta Ct}$ method was used to analyse the relative changes in the various studied mRNAs [12]. The following primers were used:
Tbp (Forward-AGGCCAGACCCCACAACTC; Reverse-GGGTGGTGCCTGGCAA),
Th (Forward-GTCTCAGAGCAGGATACCAAGC, Reverse-CTCTCCTCGAATACCACAGCC),
Dbh (Forward-GAGGCGGCTTCCATGTACG, Reverse-TCCAGGGGGATGTGGTAGG).

TH activity assay

TH enzyme activity was determined by radioenzymology according to Reinhard et al., 1986 [13]. Briefly, mouse brainstem samples were homogenized with 1.0 mL of 50 mM of 2-(N-morpholino)ethanesulfonic acid (MES) assay buffer containing 0.5% Triton X-100, pH 6.10. The supernatants (200 µL) were incubated (20 min, 37°C) with 0.8 µCi [^{3}H] L-tyrosine, 1.5 mM DL-6-methyl-5,6,7,8-tetrahydropterin (6-MPH_4), 100 U catalase and 1.0 mM dithiothreitol in a total volume of 300 µL. The reactions were terminated by adding 1.0 mL of a stirred suspension of 7.5% (w/v) charcoal in 1.0 M HCl. The mixtures were then rapidly vortexed and centrifuged at 500 x g for 10 minutes. Aliquots (200 µL) of the clear supernatant were transferred to vials containing 5 mL of scintillation cocktail and counted for tritium for quantification of [^{3}H] H_2O. Blank values, obtained by omitting 6-MPH_4, were similar to the blank values when tissue was omitted. Thus, a no 6-MPH_4 blank was routinely used. In every assay, two additional tubes, containing approximately 100,000 dpm of [^{3}H] H_2O (in 100 µL), were treated with charcoal as were the samples. The fraction of [^{3}H] H_2O recovered (generally 95%) was determined and values obtained for the remaining samples were adjusted; dividing them by this estimated recovery. The cpm were converted to pmoles/min were calculated as follows: cpm sample – cpm blank / (cpm added/2 x pmoles tyrosine added) (time) (fraction [^{3}H] H_2O recovered).

DBH activity assay

The enzymatic activity of DBH was measured according to Bouclier et al., 1977 and Cressant et al., 2017, by converting (2-^{14}C) tyramine into (2-^{14}C) octopamine, which is then subjected to periodate cleavage to form (^{14}C) *p*-hydroxybenzaldehyde [14,15]. Briefly, the brainstem was thawn, weighed, and homogenized in ten volumes of 5 mM Tris-HCl

buffer (pH 6.5) containing 0.1% Triton X-100 and was centrifuged at 15,000g for 20 minutes at 4°C. The supernatants were pipetted and used for the DBH activity assay. The reagent mixture contained the following compounds in a final volume of 1 ml: 200 µL 75 mM ascorbic acid (pH 6.0), 90 µL 17 mM pargyline, 200 µL 0.8 M sodium fumarate (pH 6), 75 µL catalase (25,000 units), 135 µL 1M acetate buffer (pH 5.5), 300 µL $CuSO_4$ under conditions appropriate to neutralize endogenous inhibitors. One volume of the cold reagent mixture was mixed with one volume of the enzyme solution (supernatants of brainstem homogenates or plasma) in a microtest tube (1.5 ml); 5 µl of this assay mixture and 5 µL of (2-^{14}C) tyramine solution (corresponding to 5.5 nmol) were transferred to a microtest tube. A brief centrifugation at 4°C allowed the two drops to be collected and mixed at the bottom of the tube. Incubation was carried out at 37°C for 0.5, 1 or 2 h depending on the enzyme activity, with the microtest tube kept closed. After incubation, the mixture was immediately placed in an ice tray and 100µl of NH_4OH 3N was added, followed by 5µl of 94mM octopamine and 10µl of 250mM tyramine. Periodate oxidation was carried out by the addition of 10 µl of 2% $NaIO_4$; 4 min later, the $NaIO_4$ excess was neutralized by the addition of 10 µl of 10% $Na_2S_20_5$. Extraction of p-hydroxybenzaldehyde: 10 µl of 40 mM EDTA and 200 µl of 5 N HCl were added to each microtest tube. After adding 1 ml of cold ethyl ether, each tube was vortexed for 30 sec and 700 µl of the organic layer was transferred to a counting bottle. A second extraction step was carried out by adding a further 900 µl of ethyl ether, mixing for 30 sec and pipetting an 800 µl aliquot of the ether layer into the counting flask. Next, 8.5ml of 0.47% omnifluor (Packard Instruments) in toluene was added to the 1.5ml extraction phase and the radioactivity was measured in a liquid scintillation counter (SL-4000, Intertechnique, France). To determine the yield of the octopamine oxidation step and the recovery of the gas phase, one volume of the reagent mixture was mixed with one volume of 10 mM Tris-HCl buffer (pH 6.5). Five µl was transferred to a microtest tube and 5 µl of 2-[^{3}H] octopamine (1.64 nmol; 11,000 cpm) was added. Incubation, oxidation and extraction of *p*-hydroxybenzaldehyde were carried out as described above. Once the specific activity of the substrate was determined, considering the percentage recovery determined with labelled octopamine corresponding to the DBH activity was calculated (94% ± 5%).

Molecular docking of *p*-cresol and *p*-cresol sulfate to TH and DBH

Molecular docking assays were performed using the HADDOCK web server based on solved protein structures of TH from *Rattus norvegicus* and human DBH (PDB identifiers: 1TOH and 4ZEL, respectively) [16–18]. For TH, residues involved in iron binding (H331, H336 and D376) were selected as active residues [18]. For DBH, residues from Cu_M and Cu_H binding sites- specifically H262, H263, H333 (Cu_M) and H412, H414, M487 (Cu_H)- were selected as active residues [16]. Passive residues surrounding the active binding sites were automatically defined using the protocol provided by the HADDOCK web server, based on structural proximity. Initial docking included rigid body minimization, followed by semi-flexible refinement, and a final stage of short molecular dynamics simulations in an explicit water solvent environment. Metal ions were included in the simulation and parametrized for the molecular dynamic step as Cu^{2+} and Fe^{3+} for DBH and TH respectively. Ligand structures were generated from SMILES code using the ELBOW tool from the Phenix suite [19,20]. Graphical representation of the best docking geometries and HADDOCK metrics for the top clusters of docking simulations for TH and DBH were then extracted. To cross-validate the results obtained with HADDOCK, molecular docking was also performed using the AutoDock Vina software via the SwissDock web server [21,22]. Structures were prepared in the same way as for HADDOCK assays.

Structural prediction of *p*-cresol and *p*-cresol sulfate binding to TH and DBH

The structures of TH (sequence from PDB ID 1TOH) and DBH (using the full-length human sequence from UniProt (ID P09172)) in complex with *p*-cresol and *p*-cresol sulfate were predicted using AlphaFold3 [23]. Both the multiple sequence alignment (MSA) and inference steps were performed using a local installation of AlphaFold.

Statistics

Data were first tested for normality using the Kolmogorov-Smirnov test. When the assumption of normality was met, comparisons between two independent groups were performed using the two-tailed Student t test while the one-sample t with the limit of detection (LOD) as a reference was used when values below the detection threshold were obtained in control groups. When normality was not met in either group, comparisons

between two independent groups were performed using the two-tailed Mann–Whitney U test. For correlation analyses, pairwise associations between variables and their corresponding p-values were assessed using Spearman's rank ρ correlation coefficient. Statistical significance was defined as $p < 0.05$ for all tests. Plotting and statistical analyses were conducted using GraphPad Prism version 8.00 (GraphPad Software, USA). A full report of statistical analyses, including normality testing, statistical test selection, group comparisons (degree of freedom, test statistic values, and p-values), and correlation details (ρ, ρ confidence intervals, and p-values) are provided either in the figure legends and/or in Supplementary Data file 1, while raw data are provided in Supplementary Data file 2.

SUPPLEMENTARY TABLES

	[*p*-Cresol]			[*p*-Cresol sulfate]			[*p*-Cresol sulfate] / [*p*-Cresol]	
Treatment	***Control***	***p-Cresol***	***Ratio p-Cresol/ Control***	***Control***	***p-Cresol***	***Ratio p-Cresol/ Control***	***Control***	***p-Cresol***
**Feces** (µmoles/g tissue)	°LOD= 0.02	0.03 ±0.01	1.58	236.67 ±52.72	428.33 ±115.70	1.81	11833.33	13442.56
**Urine** (µmoles/mg creatinine)	°LOD= 0.01	0.09 ± 0.04	9.33	5.53 ± 1.22	53.23 ±14.95	10.11	553.33	801.46
**Plasma** (nM)	°LOD= 1.00	4.00 ±2.18	4	650 ±182.80	1483.73 ±130.20	2.27	654.29	543.96 *
**Brainstem**	6.82 ±2.37 (fmol/mg tissue)	11.66 ±5.98 (fmol/mg tissue)	1.71	2298.67 ±209.60 (fmol/mg protein)	3070.67 ±207.80 (fmol/mg protein)	1.34	ND	ND

Supplementary table 1. Relationships between *p*-cresol and *p*-cresol sulfate levels across matrices and groups.
°In control feces, urine, plasma, levels were below the limit of detection (LOD).
*Two-tailed Mann-Whitney U-test: U=52, p=0.0340.
n=15/group, except for: plasmatic *p*-cresol and *p*-cresol sulfate n=14/group, brainstem *p*-cresol n=12/group.
ND: Could not be determined because the units of *p*-cresol and *p*-cresol sulfate are distinct and *p*-cresol in brainstem was measured in an independent cohort.

Reference	PMID	Metabolite	Matrix	Number of ASD	Number of NT	Method	Quantification	Effect	ASD mean concentration	NT mean concentration	Unit	ASD/NT ratio
Kang et al. 2020 mSphere	33087514	pCS	Plasma	18	20	UHPLC-MS/MS	Rel	n.s.	145469247.00	154915270.00	A.U.	0.94
Needham et al. 2020 Biol Psy	33342544	pCS	Plasma	130	92	UHPLC-MS/MS	Rel	n.s.	1.06	1.02	A.U.	1.04
Needham et al. 2020 Biol Psy	33342544	pCG	Plasma	130	92	UHPLC-MS/MS	Rel	n.s.	1.41	1.02	A.U.	1.38
Kang et al. 2020 mSphere	33087514	pCG	Plasma	15	17	UHPLC-MS/MS	Rel	n.s.	407954.00	425272.00	A.U.	0.96
De Angelis et al. 2013 PloS ONE	24130822	pC	Feces	10	10	GC - MS	Rel	↑	4.67	3.11	A.U.	1.50
Kang et al. 2020 mSphere	33087514	pC	Feces	17	18	UHPLC-MS/MS	Rel	n.s.	2691555.00	2359796.00	A.U.	1.14
Kang et al. 2018 Anaerobe	29274915	pC	Feces	23	21	^{1}H-NMR	Abs	↑	2.32	1.84	µmole per g of dry stool	1.26
Qureshi et al. 2020 J Pers Med	33023268	pC	Feces	18	20	UHPLC-MS/MS	Rel	↑	4000000.00	1550000.00	A.U.	2.58
Needham et al. 2020 Biol Psy	33342544	pCG	Feces	57	34	UHPLC-MS/MS	Rel	n.s.	1.16	0.83	A.U.	1.41
Kang et al. 2020 mSphere	33087514	pCS	Feces	18	20	UHPLC-MS/MS	Rel	↑	3995965.00	1554082.00	A.U.	2.57
Needham et al. 2020 Biol Psy	33342544	pCS	Feces	57	34	UHPLC-MS/MS	Rel	n.s.	1.18	1.70	A.U.	0.69
Gabriele et al. 2016 Autism Res	26437875	pC	Urine	53	59	HPLC- FLD	Abs	↑	138.60	91.20	µg/ml	1.52
Gevi et al. 2016 Mol Autism	27904735	pC	Urine	30	30	UHPLC-MS/MS	Rel	↑	N/A	N/A	A.U.	N/A
Mussap et al. 2020 Metabolites	33238400	pC	urine	31	26	GC-MS	Rel	↑	N/A	N/A	A.U.	2.04
Piras et al. 2022 Metabolites	35208179	pC	Urine	13	14	^{1}H-NMR	Rel	↑	20.00	6.00	A.U.	3.33
Gabriele et al. 2014 Biomarkers	25010144	pC	Urine	33	33	HPLC- FLD	Abs	n.s.	0.05	0.03	µg/ml	1.67
Altieri et al. 2011 Biomarkers	21329489	pC total (pC+pCS+pCG)	Urine	59	59	HPLC - UV–DAD	Abs	↑	123.50	91.20	µg/ml	1.35
Gabriele et al. 2014 Biomarkers	25010144	pC total (pC+pCS+pCG)	Urine	33	33	HPLC- FLD	Abs	↑	98.80	52.00	µg/ml	1.90
Gabriele et al. 2014 Biomarkers	25010144	pCG	Urine	33	33	HPLC- FLD	Abs	↑	4.80	2.70	µg/ml	1.78
Diémé et al. 2015 J Proteome Res	26538324	pCS	Urine	24	22	UHPLC-MS/MS	Rel	↑	N/A	N/A	A.U.	N/A
Nadal-Desbarats et al. 2014 Analyst	24841505	pCS	Urine	30	28	^{1}H-NMR	Abs	n.s.	96.00	90.00	µmol/mmol of creatinine	1.07
Oredskar et al. 2023 Int. J. Mol. Sci.	40806203	pCS	Urine	161	71	UHPLC-MS/MS	Abs	n.s.	56.68	52.82	µmol/mmol of creatinine	1.07
Gabriele et al. 2014 Biomarkers	25010144	pCS	Urine	33	33	HPLC- FLD	Abs	↑	92.90	49.50	µg/ml	1.88
Yap et al. 2012 J Proteome Res	20337404	pCS	Urine	39	34	^{1}H-NMR	Rel	n.s.	N/A	N/A	A.U.	N/A

Supplementary table 2. Summary of clinical studies reporting on *p*-cresol, *p*-cresol sulfate or *p*-cresol glucuronide levels in ASD patients as compared to neurotypical individuals.

Autistic (ASD), neurotypical (NT) *p*-cresol (pC), *p*-cresol sulfate (pCS), *p*-cresol glucuronide (pCS), ultra-high performance liquid chromatography tandem mass spectrometry (UHPLC-MS/MS), proton nuclear magnetic resonance (^{1}H-NMR), ultra performance liquid chromatography coupled with fluorescent detection (HPLC-FLD), ultra performance liquid chromatography coupled with ultra-violet detection (HPLC-UVD), gaz chromatography coupled with mass spectrometry (GC-MS), absolute (abs) or relative (rel) quantification of metabolite levels, effect : significant increase (↑), non significant (n.s.), data not available either in main text or in supplementary information (N/A).

Molecule	***Core structure***	***Para-substituent***
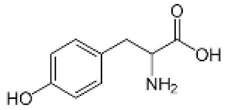 Tyrosine	Phenol ring with an amino acid side chain	-OH (hydroxyl)
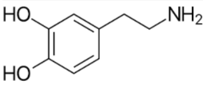 Dopamine	Catechol ring (2 -OH groups at positions 3,4)	-OH
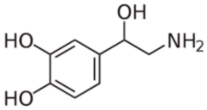 Noradrenaline	Catechol ring + β-OH side chain	-OH
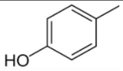 *p*-Cresol	Phenol ring with $-CH_3$ at para position	$-CH_3$
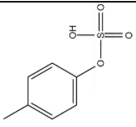 *p*-Cresol sulfate	*p*-Cresol with a sulfate group on the -OH	$-CH_3$ and $-OSO_3H$

Supplementary table 3. Structural similarities between the microbial metabolite *p*-cresol, its conjugate *p*-cresol sulfate, and endogenous substrates of TH and DBH tyrosine and dopamine.
All these molecules share a benzene ring (aromatic ring) with substituents in the para position (position 4).

		Cluster 1	Cluster 4	Cluster 3	Cluster 2	Cluster 6	Cluster 5
***p*-Cresol**	**HADDOCK score**	**-76.3 ± 1.0**	-44.4 ± 10.8	-19.9 ± 0.6	-16.8 ± 1.7	-16.2 ± 4.7	-5.2 ± 2.0
	**Cluster size**	**95**	16	19	47	4	5
	**RMSD**	**0.2 ± 0.1**	0.3 ± 0.0	0.2 ± 0.1	0.4 ± 0.0	0.3 ± 0.0	0.4 ± 0.1
	**Van der Waals energy**	**48.6 ± 2.7**	8.5 ± 29.4	-9.6 ± 0.6	-6.3 ± 0.5	-8.3 ± 3.1	-3.7 ± 1.8
	**Electrostatic energy**	**-599.4 ± 14.8**	-257.2 ± 197.2	-21.0 ± 6.8	-27.9 ± 16.3	-16.6 ± 8.9	-24.8 ± 15.0
	**Desolvation energy**	**-5.1 ± 1.1**	-1.7 ± 0.8	-6.6 ± 1.4	-5.3 ± 1.9	-5.0 ± 0.7	-0.3 ± 1.2
	**Restraints violation energy**	**1.3 ± 0.5**	2.3 ± 1.4	5.0 ± 0.6	4.5 ± 0.8	3.6 ± 2.9	38.4 ± 5.0
	**Buried Surface Area**	**400.9 ± 6.1**	407.6 ± 12.5	367.7 ± 11.1	298.0 ± 7.6	341.5 ± 14.9	170.9 ± 22.8
	**Z-Score**	**-1.9**	-0.6	0.4	0.5	0.6	1
		**Cluster 1**	**Cluster 3**	**Cluster 4**	**Cluster 2**	**Cluster 6**	**Cluster 5**
***p*-Cresol sulfate**	**HADDOCK score**	**-20.2 ± 0.5**	-18.9 ± 0.6	-18.0 ± 0.3	-13.6 ± 0.2	-13.2 ± 0.8	-7.3 ± 2.0
	**Cluster size**	**107**	19	19	28	5	5
	**RMSD**	**0.2 ± 0.1**	0.3 ± 0.0	0.4 ± 0.1	0.3 ± 0.0	0.3 ± 0.0	0.4 ± 0.1
	**Van der Waals energy**	**-11.5 ± 2.9**	-11.7 ± 0.3	-12.3 ± 1.3	-7.1 ± 0.7	-7.9 ± 0.8	-6.8 ± 2.1
	**Electrostatic energy**	**-11.1 ± 15.0**	-0.7 ± 1.2	-5.1 ± 6.7	0.7 ± 1.1	0.3 ± 0.7	-0.9 ± 0.9
	**Desolvation energy**	**-6.9 ± 1.0**	-7.2 ± 0.5	-4.8 ± 1.0	-6.9 ± 0.6	-5.7 ± 0.9	-0.8 ± 1.0
	**Restraints violation energy**	**3.3 ± 0.8**	1.5 ± 0.4	1.7 ± 0.8	2.3 ± 0.9	3.4 ± 1.3	5.6 ± 1.8
	**Buried Surface Area**	**320.6 ± 9.4**	315.2 ± 6.5	318.4 ± 5.1	272.8 ± 12.5	260.9 ± 17.8	249.3 ± 11.6
	**Z-Score**	**-1.1**	-0.8	-0.6	0.4	0.5	1.8

Supplementary table 4. Key metrics for *p*-cresol and *p*-cresol sulfate HADDOCK docking clusters in TH.
Summary of the top docking metrics for *p*-cresol and *p*-cresol sulfate binding to TH obtained from HADDOCK. For *p*-cresol, Cluster 1 ranks highest, displaying the most favorable HADDOCK score (−76.3 ± 1.0) and the smallest root mean square deviation of atomic positions (RMSD) from the overall lowest-energy structure (0.2 ± 0.1 Å). It also exhibits significantly much stronger van der Waals energy and electrostatic energy scores compared to other clusters. Similarly, for *p*-cresol sulfate, Cluster 1 shows the best HADDOCK score (-20.2 ± 0.5) and lowest RMSD (0.2 ± 0.1). Clusters 3 and 4 follow, with comparable van der Waals energies, but less favorable electrostatic interactions. Notably, structural inspection reveals that all 3 clusters position *p*-cresol sulfate within the catalytic pocket of TH.

	p-Cresol			*p*-Cresol sulfate			
	**Cluster 1**	**Cluster 3**	**Cluster 2**	**Cluster 1**	**Cluster 2**	**Cluster 4**	**Cluster 3**
**HADDOCK score**	-68.1 ± 2.0	-35.7 ± 17.4	-32.6 ± 0.7	-23.0 ± 0.6	-19.8 ± 0.8	-17.1 ± 2.6	-14.1 ± 1.8
**Cluster size**	179	8	10	175	10	4	6
**RSMD**	0.2 ± 0.1	0.2 ± 0.0	0.3 ± 0.1	0.2 ± 0.1	0.6 ± 0.3	0.4 ± 0.1	0.3 ± 0.0
**Van der Waals energy**	18.7 ± 4.8	-13.3 ± 2.6	-1.7 ± 2.5	-13.3 ± 1.1	-13.7 ± 0.5	-11.9 ± 3.1	-10.3 ± 1.2
**Electrostatic energy**	-417.4 ± 20.2	-90.1 ± 101.3	-148.0 ± 18.0	-7.1 ± 7.4	-0.2 ± 1.7	-0.6 ± 1.7	0.5 ± 1.9
**Desolvation energy**	-3.6 ± 0.5	-4.8 ± 0.8	-1.6 ± 1.3	-8.5 ± 1.8	-6.3 ± 0.2	-5.2 ± 0.6	-4.0 ± 0.3
**Restraints violation energy**	2.9 ± 2.5	4.5 ± 2.6	3.1 ± 1.9	1.5 ± 1.0	1.9 ± 1.1	1.7 ± 1.1	1.2 ± 0.7
**Buried Surface Area**	414.8 ± 5.3	406.4 ± 11.4	412.3 ± 23.2	335.4 ± 18.2	345.4 ± 5.6	344.9 ± 16.8	277.6 ± 17.7
**Z-Score**	-1.4	0.6	0.8	-1.4	-0.4	0.4	1.3

Supplementary table 5. Key metrics for *p*-cresol and *p*-cresol sulfate HADDOCK docking clusters in DBH.
Summary of the top docking metrics for *p*-cresol and *p*-cresol sulfate binding to DBH obtained from HADDOCK. For *p*-cresol, Cluster 1 ranks highest, displaying the most favorable HADDOCK score (−61.8 ± 2.0) and the smallest RSMD (0.2 ± 0.1 Å). It also shows a markedly stronger electrostatic energy score and moderately improved van der Waals energy compared to other clusters. Similarly, for *p*-cresol sulfate, Cluster 1 also ranks highest, with the most favorable HADDOCK score (−23.2 ± 0.6) and the smallest RMSD (0.2 ± 0.1 Å). However, Clusters 2, 3, and 4 display comparable docking scores, indicating closely related binding affinities. Structural inspection reveals that all top-ranking clusters for *p*-cresol sulfate localize the ligand within the catalytic pocket of DBH. The docking geometries consistently show the sulfate group coordinating with the copper ion, while the tyrosyl moiety engages in π-stacking interactions with adjacent aromatic residues.

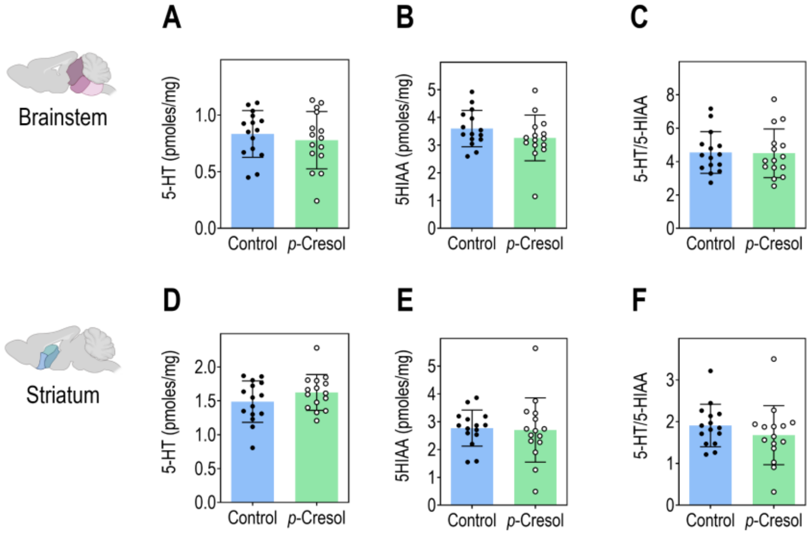

Supplementary figure 1. *p*-Cresol exposure does not impact the serotoninergic system in brainstem and striatum.
A. Serotonin (5-HT) levels in brainstem. Student t test: t=0.6506, df=28, p=0.5206.
B. 5-Hydroxyindoleacetic acid (5-HIAA) levels in brainstem. Student t test: t=1.242, df=28, p=0.2245.
C. 5-HT turnover: ratio of 5-HT and 5-HIAA levels in brainstem. Student t test: t=0.09564, df=28, p=0.9245.
D. Serotonin (5-HT) levels in striatum. Student t test: t=1.299, df=28, p=0.2045.
E. 5-HIAA levels in striatum. Student t test: t=0.1961, df=28, p=0.846.
F. 5-HT turnover: 5-HT/5-HIAA ratio in striatum. Student t test: t=1.028, df=28, p=0.3129.
(A-F) n=15/group. Data are presented as dot plots showing means ± standard deviation.

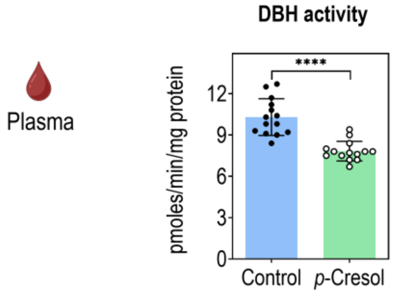

Supplementary figure 2. *p*-Cresol exposure decreases plasma DBH activity.
Dopamine-β-hydroxylase activity (DBH) in plasma (n= 14/group). Mann-Whitney U test: U=7, ****p<0.0001. Data are presented as dot plots showing means ± standard deviation.

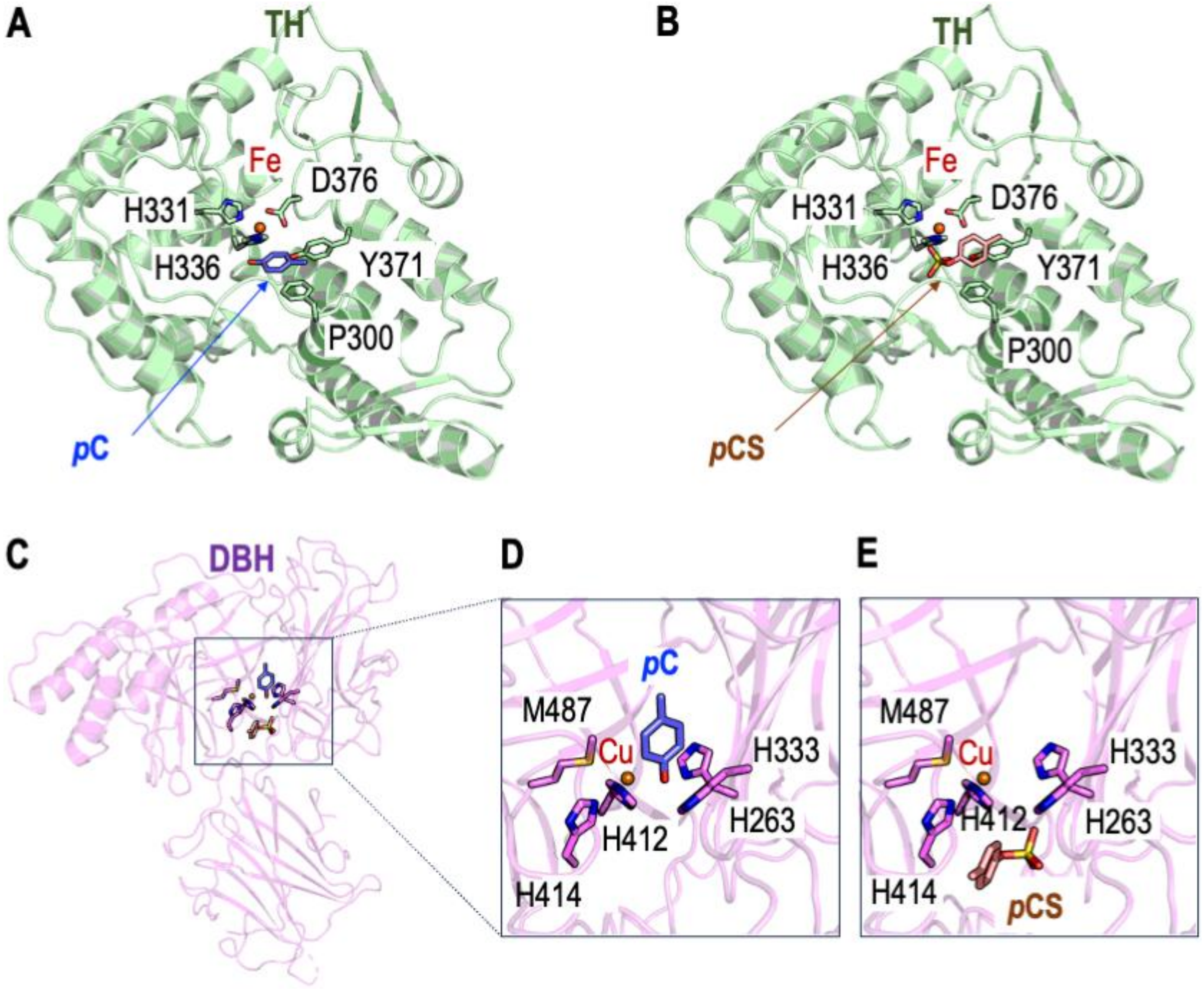

Supplementary figure 3. Optimal docking geometries of *p*-cresol and *p*-cresol sulfate within TH and DBH active sites predicted by AutoDock Vina.

A. Predicted docking geometry of *p*-cresol (*p*C, in blue) within the active site of tyrosine hydroxylase (TH, in green), showing interaction near the catalytic iron ion (Fe, in red).

B. Predicted docking geometry of *p*-cresol sulfate (*p*CS, in orange) within the TH active site (in green), positioned near the catalytic iron ion (Fe, in red).

C. Overview of docking geometries showing superimposed binding poses of *p*-cresol and *p*-cresol sulfate in complex with DBH (in purple).

D. Close-up view of the optimal binding pose of *p*-cresol (*p*C, in blue) within the DBH active site, showing proximity to the copper ion (Cu, in red).

E. Close-up view of the optimal binding pose of *p*-cresol sulfate (*p*CS, in orange) within the DBH active site, highlighting interaction with the catalytic copper ion (Cu, in red).

.

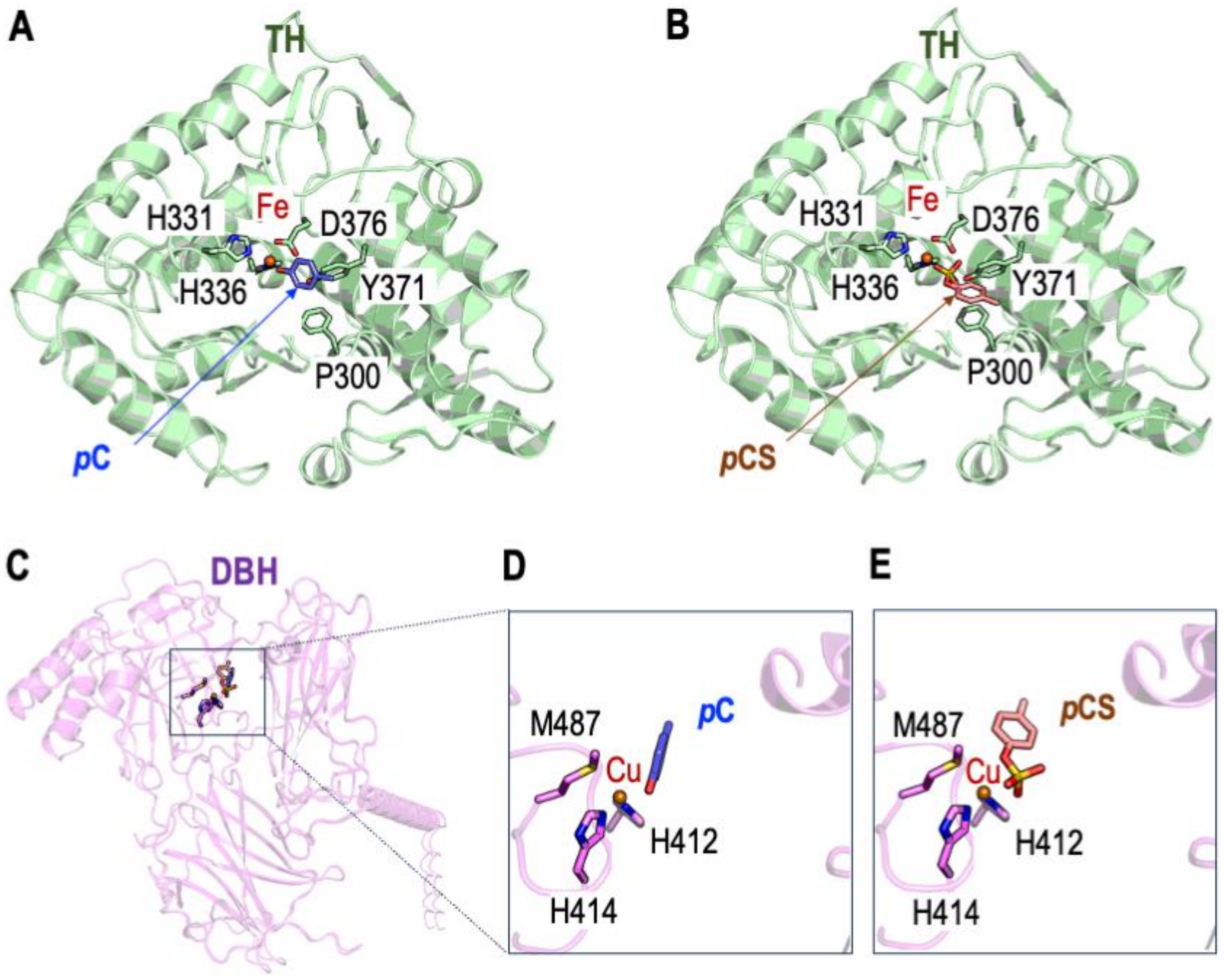

Supplementary figure 4. Structural predictions of *p*-cresol and *p*-cresyl sulfate bound to TH and DBH, based on AlfaFold3 modeling.

A. High-confidence predicted structure for TH (in green) in complex with *p*-cresol (*p*C, in blue), within the active site, showing interaction near the catalytic iron ion (Fe, in red).

B. High-confidence predicted structure for TH (in green) in complex with *p*-cresol sulfate (*pCS*, in orange), within the active site, showing interaction near the catalytic iron ion (Fe, in red).

C. Overview of high-confidence predicted structure for DBH (in purple) showing superimposed binding poses of *p*-cresol and *p*-cresol sulfate.

D. Close-up view of the high-confidence predicted structure for DBH complexed with *p*-cresol (*p*C, in blue), within the active site, showing interaction near the catalytic copper ion (Cu, in red).

E. Close-up view of the high-confidence predicted structure for DBH complexed with *p*-cresol sufate (*p*CS, in orange), within the active site, showing interaction near the catalytic copper ion (Cu, in red).

REFERENCES

1. Loomes, R., Hull, L. & Mandy, W. P. L. What Is the Male-to-Female Ratio in Autism Spectrum Disorder? A Systematic Review and Meta-Analysis. *J. Am. Acad. Child Adolesc. Psychiatry* **56**, 466–474 (2017).
2. Bermudez-Martin, P. *et al.* The microbial metabolite p-Cresol induces autistic-like behaviors in mice by remodeling the gut microbiota. *Microbiome* **9**, 1–23 (2021).
3. Acosta, M. C., Tillage, R. P., Weinshenker, D. & Saltzman, W. Acute inhibition of dopamine β-hydroxylase attenuates behavioral responses to pups in adult virgin California mice (Peromyscus californicus). *Horm. Behav.* **137**, 105086 (2022).
4. Loureiro, A. I. *et al.* Role of P-glycoprotein and permeability upon the brain distribution and pharmacodynamics of etamicastat: a comparison with nepicastat. *Xenobiotica* **45**, 828–839 (2015).
5. Schroeder, J. P., Alisha Epps, S., Grice, T. W. & Weinshenker, D. The Selective Dopamine β-Hydroxylase Inhibitor Nepicastat Attenuates Multiple Aspects of Cocaine-Seeking Behavior. *Neuropsychopharmacol. 2013 386* **38**, 1032–1038 (2013).
6. Gao, X. *et al.* Metabolite analysis of human fecal water by gas chromatography/mass spectrometry with ethyl chloroformate derivatization. *Anal. Biochem.* **393**, 163–175 (2009).
7. Korytowska, N., Wyczałkowska-Tomasik, A., Wiśniewska, A., Pączek, L. & Giebułtowicz, J. Development of the LC-MS/MS method for determining the p-cresol level in plasma. *J. Pharm. Biomed. Anal.* **167**, 149–154 (2019).
8. Santa, T. Derivatization reagents in liquid chromatography/electrospray ionization tandem mass spectrometry. *Biomed. Chromatogr.* **25**, 1–10 (2011).
9. Fabregat-Safont, D. *et al.* Improving the detectability of low-abundance p-cresol in biological matrices by chemical derivatization and LC-MS/MS determination. *Talanta* 127770 (2025) doi:10.1016/j.talanta.2025.127770.
10. Monošík, R. & Dragsted, L. O. A versatile UHPLC-MSMS method for simultaneous quantification of various alcohol intake related compounds in human urine and blood. *Anal. Methods* **8**, 6865–6871 (2016).
11. Kema, I. P. *et al.* High performance liquid chromatographic profiling of tryptophan and related indoles in body fluids and tissues of carcinoid patients. *Clin. Chim. Acta* **221**, 143–158 (1993).
12. Livak, K. J. & Schmittgen, T. D. Analysis of Relative Gene Expression Data Using Real-Time Quantitative PCR and the 2−ΔΔCT Method. *Methods* **25**, 402–408 (2001).
13. Reinhard, J. F., Smith, G. K. & Nichol, C. A. A rapid and sensitive assay for tyrosine-3-monooxygenase based upon the release of 3H2O and adsorption of [3H]-tyrosine by charcoal. *Life Sci.* **39**, 2185–2189 (1986).
14. Bouclier, M., Mandel, P. & Aunis, D. Microdetermination of dopamine-β-hydroxylase activities using a simplified, single step radioenzymatic assay. *Pharmacol. Res. Commun.* **9**, 743–754 (1977).
15. Cressant, A. *et al.* Loss-of-function of PTPR γ and ζ, observed in sporadic schizophrenia, causes brain region-specific deregulation of monoamine levels and altered behavior in mice. *Psychopharmacology (Berl).* **234**, 575–587 (2017).

16. Vendelboe, T. V. *et al.* The crystal structure of human dopamine β-hydroxylase at 2.9 Å resolution. *Sci. Adv.* **2**, (2016).
17. Honorato, R. V. *et al.* The HADDOCK2.4 web server for integrative modeling of biomolecular complexes. *Nat. Protoc. 2024 1911* **19**, 3219–3241 (2024).
18. Goodwill, K. E. *et al.* Crystal structure of tyrosine hydroxylase at 2.3 Å and its implications for inherited neurodegenerative diseases. *Nat. Struct. Biol. 1997 47* **4**, 578–585 (1997).
19. Liebschner, D. *et al.* Macromolecular structure determination using X-rays, neutrons and electrons: recent developments in Phenix. *urn:issn:2059-7983* **75**, 861–877 (2019).
20. Moriarty, N. W., Grosse-Kunstleve, R. W. & Adams, P. D. electronic Ligand Builder and Optimization Workbench (eLBOW): a tool for ligand coordinate and restraint generation. *urn:issn:0907-4449* **65**, 1074–1080 (2009).
21. Bugnon, M. *et al.* SwissDock 2024: major enhancements for small-molecule docking with Attracting Cavities and AutoDock Vina. *Nucleic Acids Res.* **52**, W324–W332 (2024).
22. Eberhardt, J., Santos-Martins, D., Tillack, A. F. & Forli, S. AutoDock Vina 1.2.0: New Docking Methods, Expanded Force Field, and Python Bindings. *J. Chem. Inf. Model.* **61**, 3891–3898 (2021).
23. Abramson, J. *et al.* Accurate structure prediction of biomolecular interactions with AlphaFold 3. *Nat. 2024 6308016* **630**, 493–500 (2024).
