# Supplementary material for: The autism-linked gut microbial metabolite p-cresol inhibits host catecholamine biosynthesizing enzymes to elicit social deficits
Source: Commun Biol. 2025 Nov 22;8:1800. doi: 10.1038/s42003-025-09207-0 (PMC12722727; doi:10.1038/s42003-025-09207-0)
Supplement: Supplementary file 2 — Description of additional supplementary files [file 42003_2025_9207_MOESM2_ESM.docx]

Description of additional supplementary file

File name: Supplementary Data 1

Description: full report of statistical analyses, including sample sizes, normality testing, statistical test selection, group comparisons (degree of freedom, test statistic values, and p-values), and correlation details (ρ, ρ confidence intervals, and p-values)

File name: Supplementary Data 2

Description: raw data corresponding to figures from the manuscript
