# Supplementary material for: The autism-linked gut microbial metabolite p-cresol inhibits host catecholamine biosynthesizing enzymes to elicit social deficits
Source: Commun Biol. 2025 Nov 22;8:1800. doi: 10.1038/s42003-025-09207-0 (PMC12722727; doi:10.1038/s42003-025-09207-0)
Supplement: Supplementary file 5 — Reporting Summary [file 42003_2025_9207_MOESM5_ESM.pdf]

nature portfolio

Corresponding author(s): Laetitia Davidovic davidovic@ipmc.cnrs.fr

Last updated by author(s): Nov 3, 2025

Reporting Summary

Nature Portfolio wishes to improve the reproducibility of the work that we publish. This form provides structure for consistency and transparency in reporting. For further information on Nature Portfolio policies, see our Editorial Policies and the Editorial Policy Checklist.

Statistics

For all statistical analyses, confirm that the following items are present in the figure legend, table legend, main text, or Methods section.

n/a	Confirmed	
☐	☒	The exact sample size (*n*) for each experimental group/condition, given as a discrete number and unit of measurement
☐	☒	A statement on whether measurements were taken from distinct samples or whether the same sample was measured repeatedly
☐	☒	The statistical test(s) used AND whether they are one- or two-sided<br>*Only common tests should be described solely by name; describe more complex techniques in the Methods section.*
☐	☒	A description of all covariates tested
☐	☒	A description of any assumptions or corrections, such as tests of normality and adjustment for multiple comparisons
☐	☒	A full description of the statistical parameters including central tendency (e.g. means) or other basic estimates (e.g. regression coefficient) AND variation (e.g. standard deviation) or associated estimates of uncertainty (e.g. confidence intervals)
☐	☒	For null hypothesis testing, the test statistic (e.g. *F*, *t*, *r*) with confidence intervals, effect sizes, degrees of freedom and *P* value noted<br>*Give P values as exact values whenever suitable.*
☒	☐	For Bayesian analysis, information on the choice of priors and Markov chain Monte Carlo settings
☒	☐	For hierarchical and complex designs, identification of the appropriate level for tests and full reporting of outcomes
☒	☐	Estimates of effect sizes (e.g. Cohen's *d*, Pearson's *r*), indicating how they were calculated

Our web collection on statistics for biologists contains articles on many of the points above.

Software and code

Policy information about availability of computer code

Data collection	no software were used
Data analysis	Molecular docking assays were performed using the HADDOCK web server based on solved protein structures of TH from Rattus norvegicus and human DBH (PDB identifiers: 1TOH and 4ZEL, respectively) as detailed in the Supplementary Methods. Ligand structures were generated from SMILES code using the ELBOW tool from the Phenix suite . To cross-validate the results obtained with HADDOCK, molecular docking was also performed using the AutoDock Vina software via the SwissDock web server. The structures of TH (amino-acid sequence PDB identifier: 1TOH) and DBH (using the full-length human amino-acid sequence (UniProt identifier: P09172)) in complex with p-cresol and p-cresol sulfate were predicted using AlphaFold3. Both the multiple sequence alignment (MSA) and inference steps were performed using a local installation of AlphaFold. Plotting and statistical analyses were conducted using GraphPad Prism version 8.00 (GraphPad Software, USA)

For manuscripts utilizing custom algorithms or software that are central to the research but not yet described in published literature, software must be made available to editors and reviewers. We strongly encourage code deposition in a community repository (e.g. GitHub). See the Nature Portfolio guidelines for submitting code & software for further information.

Data

Policy information about availability of data

All manuscripts must include a data availability statement. This statement should provide the following information, where applicable:

- Accession codes, unique identifiers, or web links for publicly available datasets
- A description of any restrictions on data availability
- For clinical datasets or third party data, please ensure that the statement adheres to our policy

Detailed report of statistics (normality of data, detailed statistics for group comparison and correlations) are provided in Supplementary Data 1. The datasets generated and analyzed during the current study are provided in Supplementary Data 2.

Research involving human participants, their data, or biological material

Policy information about studies with human participants or human data. See also policy information about sex, gender (identity/presentation), and sexual orientation and race, ethnicity and racism.

Reporting on sex and gender	*Use the terms sex (biological attribute) and gender (shaped by social and cultural circumstances) carefully in order to avoid confusing both terms. Indicate if findings apply to only one sex or gender; describe whether sex and gender were considered in study design; whether sex and/or gender was determined based on self-reporting or assigned and methods used. Provide in the source data disaggregated sex and gender data, where this information has been collected, and if consent has been obtained for sharing of individual-level data; provide overall numbers in this Reporting Summary. Please state if this information has not been collected. Report sex- and gender-based analyses where performed, justify reasons for lack of sex- and gender-based analysis.*
Reporting on race, ethnicity, or other socially relevant groupings	*Please specify the socially constructed or socially relevant categorization variable(s) used in your manuscript and explain why they were used. Please note that such variables should not be used as proxies for other socially constructed/relevant variables (for example, race or ethnicity should not be used as a proxy for socioeconomic status). Provide clear definitions of the relevant terms used, how they were provided (by the participants/respondents, the researchers, or third parties), and the method(s) used to classify people into the different categories (e.g. self-report, census or administrative data, social media data, etc.) Please provide details about how you controlled for confounding variables in your analyses.*
Population characteristics	*Describe the covariate-relevant population characteristics of the human research participants (e.g. age, genotypic information, past and current diagnosis and treatment categories). If you filled out the behavioural & social sciences study design questions and have nothing to add here, write "See above."*
Recruitment	*Describe how participants were recruited. Outline any potential self-selection bias or other biases that may be present and how these are likely to impact results.*
Ethics oversight	*Identify the organization(s) that approved the study protocol.*

Note that full information on the approval of the study protocol must also be provided in the manuscript.

Field-specific reporting

Please select the one below that is the best fit for your research. If you are not sure, read the appropriate sections before making your selection.

☒ Life sciences ☐ Behavioural & social sciences ☐ Ecological, evolutionary & environmental sciences

For a reference copy of the document with all sections, see nature.com/documents/nr-reporting-summary-flat.pdf

Life sciences study design

All studies must disclose on these points even when the disclosure is negative.

Sample size	The study was conducted in accordance with procedures approved by the local ethics committee for animal experimentation (Ciepal-Azur) and the Ministère de l'Enseignement Supérieur et de la Recherche (APAFIS), in agreement with the European Communities Council Directive (2010/63EU) for animal experiments (Agreements references: APAFIS #21355-2019062414391395 v3, APAFIS #51032-2024082612133352 v5).
Data exclusions	no data were excluded
Replication	We have previously described that this treatment was not interfering with basic physiological parameters (drink/food consumption, body weight) but led to social interaction deficits, with no impact on locomotor activity, exploratory behavior, anxiety or cognition (Bermudez et al., 2021).
Randomization	Upon arrival, animals were randomly allocated to the experimental groups and housed in cages of 4 to 5 animals

Blinding	Blinding location were not possible. But Each recording session included four individual animals—two controls (or vehicle-treated) and two treated—randomly assigned to the four simultaneously monitored open fields. Manual scoring by an experienced experimenter blind to the experimental group was performed a posteriori by recording the number of nose contacts and time spent in nose contact, as previously (Bermudez et al;, 2021).

Reporting for specific materials, systems and methods

We require information from authors about some types of materials, experimental systems and methods used in many studies. Here, indicate whether each material, system or method listed is relevant to your study. If you are not sure if a list item applies to your research, read the appropriate section before selecting a response.

Materials & experimental systems

n/a	Involved in the study
☒	☐ Antibodies
☒	☐ Eukaryotic cell lines
☒	☐ Palaeontology and archaeology
☐	☒ Animals and other organisms
☒	☐ Clinical data
☒	☐ Dual use research of concern
☒	☐ Plants

Methods

n/a	Involved in the study
☒	☐ ChIP-seq
☒	☐ Flow cytometry
☒	☐ MRI-based neuroimaging

Animals and other research organisms

Policy information about studies involving animals; ARRIVE guidelines recommended for reporting animal research, and Sex and Gender in Research

Laboratory animals	Weaned and 9-weeks oldC57BL/6J (authenticated Jax® mouse strain) male mice (21 to 28 days old) were obtained from Charles River (France)
Wild animals	the study did not involved wild animals
Reporting on sex	Only males were considered in this study, as the ASD sex ratio is biased towards 3 males diagnosed for 1 female, according to
Field-collected samples	the study did not involve collect on the field.
Ethics oversight	Animal housing and experimentation were conducted in facilities certified by regional authorities (Direction Départementale de Protection des Populations des Alpes-Maritimes, accreditation #C-06-152-5). The study was conducted in accordance with procedures approved by the local ethics committee for animal experimentation (Ciepal-Azur) and the Ministère de l'Enseignement Supérieur et de la Recherche (APAFIS), in agreement with the European Communities Council Directive (2010/63EU) for animal experiments (Agreements references: APAFIS #21355-2019062414391395 v3, APAFIS #51032-2024082612133352 v5).

Note that full information on the approval of the study protocol must also be provided in the manuscript.

Plants

Seed stocks	*Report on the source of all seed stocks or other plant material used. If applicable, state the seed stock centre and catalogue number. If plant specimens were collected from the field, describe the collection location, date and sampling procedures.*
Novel plant genotypes	*Describe the methods by which all novel plant genotypes were produced. This includes those generated by transgenic approaches, gene editing, chemical/radiation-based mutagenesis and hybridization. For transgenic lines, describe the transformation method, the number of independent lines analyzed and the generation upon which experiments were performed. For gene-edited lines, describe the editor used, the endogenous sequence targeted for editing, the targeting guide RNA sequence (if applicable) and how the editor was applied.*
Authentication	*Describe any authentication procedures for each seed stock used or novel genotype generated. Describe any experiments used to assess the effect of a mutation and, where applicable, how potential secondary effects (e.g. second site T-DNA insertions, mosiacism, off-target gene editing) were examined.*
